# Mitochondrial Bioenergetics of Functional Wound Closure is Dependent on Macrophage–Keratinocyte Exosomal Crosstalk

**DOI:** 10.1021/acsnano.4c07610

**Published:** 2024-10-25

**Authors:** Anu Sharma, Rajneesh Srivastava, Surya C. Gnyawali, Pramod Bhasme, Adam J. Anthony, Yi Xuan, Jonathan C. Trinidad, Chandan K. Sen, David E. Clemmer, Sashwati Roy, Subhadip Ghatak

**Affiliations:** † McGowan Institute for Regenerative Medicine, Department of Surgery, University of Pittsburgh, Pittsburgh, Pennsylvania 15219, United States; ‡ Department of Chemistry, Indiana University, Bloomington, Indiana 47405, United States

**Keywords:** macrophage-derived exosomes, TOMM70, keratinocyte migration, functional wound closure, macrophage–keratinocyte crosstalk, tissue nanotransfection, “don’t eat me” plasmid

## Abstract

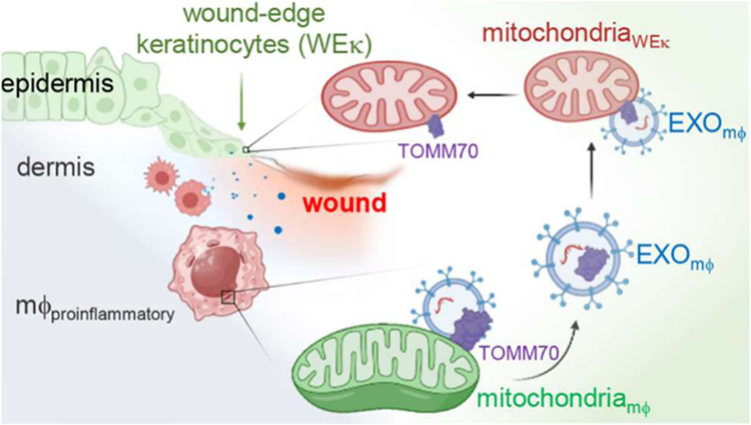

Tissue nanotransfection (TNT)-based fluorescent labeling of cell-specific exosomes has shown that exosomes play a central role in physiological keratinocyte–macrophage (mϕ) crosstalk at the wound-site. Here, we report that during the early phase of wound reepithelialization, macrophage-derived exosomes (Exo_mϕ_), enriched with the outer mitochondrial membrane protein TOMM70, are localized in leading-edge keratinocytes. TOMM70 is a 70 kDa adaptor protein anchored in the mitochondrial outer membrane and plays a critical role in maintaining mitochondrial function and quality. TOMM70 selectively recognizes cytosolic chaperones by its tetratricopeptide repeat (TPR) domain and facilitates the import of preproteins lacking a positively charged mitochondrial targeted sequence. Exosomal packaging of TOMM70 in mϕ was independent of mitochondrial fission. TOMM70-enriched Exo_mϕ_ compensated for the hypoxia-induced depletion of epidermal TOMM70, thereby rescuing mitochondrial metabolism in leading-edge keratinocytes. Thus, macrophage-derived TOMM70 is responsible for the glycolytic ATP supply to power keratinocyte migration. Blockade of exosomal uptake from keratinocytes impaired wound closure with the persistence of proinflammatory mϕ in the wound microenvironment, pointing toward a bidirectional crosstalk between these two cell types. The significance of such bidirectional crosstalk was established by the observation that in patients with nonhealing diabetic foot ulcers, TOMM70 is deficient in keratinocytes of wound-edge tissues.

Wound healing and tissue repair necessitate dynamic metabolic adaptations that coordinate intrinsic and extrinsic cellular responses.^1^ Inflammatory macrophages (mϕ) rely on glycolysis, generating lactate and succinate, which serve as signaling molecules influencing inflammatory responses.^2,3^ Succinate stabilizes HIF-1α, thus enhancing acute inflammation, while lactate promotes reparative functions through histone lactylation and angiogenesis.^4−6^ In the dynamic microenvironment of the injured cutaneous tissue, cellular constituents undergo substantial metabolic reprogramming throughout the course of the healing trajectory. This necessitates intricate intercellular communication to balance energy requirements. Resident cells communicate among themselves through gap junctions or tunneling nanotubes transporting subcellular organelles such as mitochondria.^7^ This concept of intercellular energy synchronization, although reported nearly two decades ago,^8,9^ has garnered renewed interest in recent years, particularly in the context of paracrine transport of mitochondrial parts or in its entirety in tissue repair. Mitochondria have the unique ability to traverse cellular boundaries, actively participating in intercellular crosstalk either through juxtacrine or paracrine mechanisms.^10,11^ This concept of “mitochondrial altruism” has been demonstrated previously.^12−14^ The transfer of extracellular intact mitochondria from astrocytes to neurons following injury has been shown to promote ATP production and viability, thereby offering a form of neuroprotection.^15^ The transfer of polarized mitochondria between human brain endothelial cells to neurons bolsters cellular energetics in ischemic endothelial cells.^16^ Paracrine transfer of mitochondrial cargo occurs under physiological and pathological conditions such as hypoxia and inflammation.^12^ Such transfer, arguably via mϕ, has been reported at the inflammatory phase post injury.^17^

Reports from our and other laboratories support the notion of bidirectional paracrine signaling between resident keratinocytes and blood-borne myeloid cells recruited to the wound microenvironment.^18−22^ At the wound-site, blood-borne macrophages (ωmϕ) must mount and resolve inflammation in a timely manner to enable physiological tissue repair. Bidirectional paracrine crosstalk between wound-site keratinocytes and mϕ is necessary.^19^ Following injury, the keratinocytes generate signaling molecules that prompt mϕ to come to the injury site to counter the invading pathogens or infection, helping to reduce inflammation and reshape tissues.^23^ Conversely, the mϕ affect the functions of keratinocytes by releasing cytokines and growth factors that influence their proliferation and migration to achieve functional wound closure.^23,24^ Effective communication in both directions is crucial for the healing process,^25,26^ and any disruption in this signaling process can result in chronic nonhealing wounds or cutaneous inflammatory issues, as observed in psoriasis.^27,28^ In this work, using cell-specific exosome labeling and isolation techniques, we have unraveled that proinflammatory mϕ selectively packages a mitochondrial part, the outer mitochondrial protein TOMM70, into exosomes. TOMM70 is hypoxia-sensitive and drives keratinocyte migration, which is necessary for reepithelialization. Exosomal packaging of TOMM70 in wound-site mϕ is guided by cues from wound-edge (WE) keratinocytes. Any failure of this mechanism hinders keratinocyte migration and impairs functional wound closure.

## Results and Discussion

### Wound-Edge Tissue Segmentation Identified Exosome-Mediated Keratinocyte-mϕ Bidirectional Crosstalk

**Figure 1:**
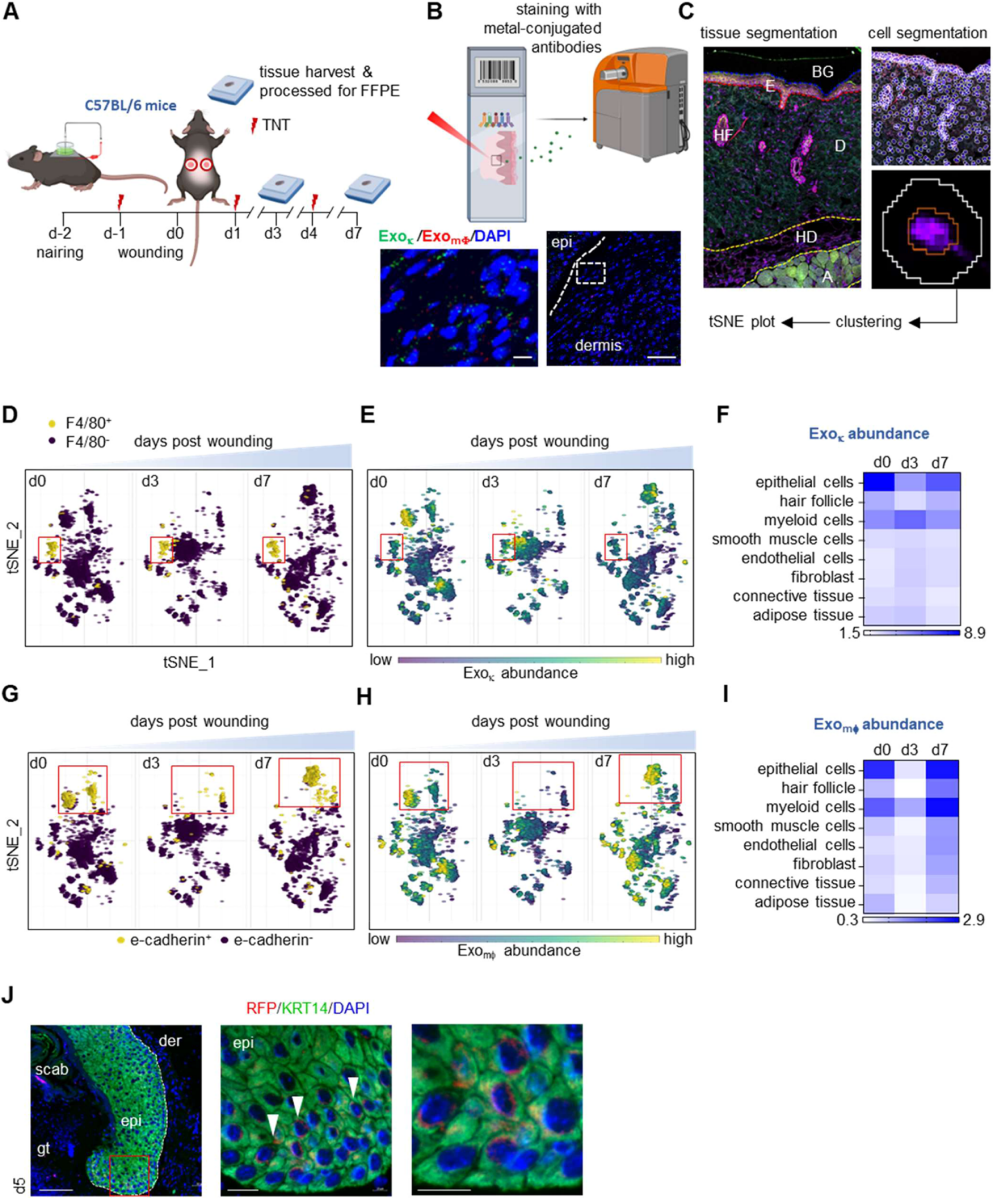
Application of a deep learning algorithm for tissue segmentation in WE tissue postimaging mass cytometry identified keratinocyte-mϕ crosstalk as the predominant exosome-mediated paracrine signaling. (A) Schematic diagram showing delivery of plasmid cocktails in WE tissue of C57BL/6 mice at different time points. (B) Schematic diagram showing the staining of murine WE tissue with metal-conjugated antibodies for Imaging Mass Cytometry. Localization of Exo_κ_ and Exo_mϕ_ in dermis d3 post-TNT. Scale, 100 and 10 μm. (C) Deep learning algorithm-based marker antibodies were used for tissue segmentation. Based on the segmentation, the skin and WE tissue were divided into six segments: background (BG), hair follicles (HF), epidermis (E), dermis (D), hypodermis (HD), and adipose tissue (A). A deep learning classifier based on DNA1 and DNA2 channels with expansion of the cell body from the nuclei was used for cell segmentation. The white circle indicates the entire cell, and the red circle demonstrates nuclear compartments. (D) t-SNE plot with a binary color bar showing an abundance of F4/80 cell distributions at d0, d3, and d7 post wounding. (E) Abundance of Exo_κ_ in F4/80^+^ cells was plotted on a tSNE plot with a continuous color bar. (F) Heat map showing the distribution of Exo_κ_ in different cell types at d0, d3, and d7 post wounding. (G) t-SNE plot with a binary color bar showing an abundance of e-cadherin^+^ cell distributions at d0, d3, and d7 post wounding. (H) Abundance of Exo_mϕ_ in e-cadherin^+^ cells was plotted on a t-SNE plot with a continuous color bar. (I) Heat map showing the distribution of Exo_mϕ_ in different cell types at d0, d3, and d7 post wounding. (J) Representative high-resolution confocal microscopic images demonstrating the presence of Exo_mϕ_ (red) in keratinocytes (green) in murine d5 WE tissue. Scale, 100, 20, and 10 μm.

Exosome-mediated intercellular communication was studied at the WE using a cocktail of two promoter-driven plasmids encoding CD9, CD63, and CD81 with an “in-frame” reporter sequence. The first set of plasmids was driven by a *Krt14* promoter with a GFP reporter. Promoter specificity and reporter expression have been reported previously.^19,29^ The second set of plasmids was driven by the *Lyz2* promoter with an RFP reporter protein of *Discosoma* origin (phylum Cnidaria; not expressed in vertebrates) (Figure S1A). The promoter specificity of the plasmid cocktail was tested in mouse keratinocytes, mϕ, endothelial cells, and fibroblasts (Figure S1B,C). The plasmid cocktail was delivered on the mouse dorsum via tissue nanotransfection (TNT) (Figures 1A and S1D). The presence of macrophage-derived macrophages (Exo_mϕ_) with an RFP reporter and keratinocyte-derived exosomes (Exo_κ_) with GFP reporter expression was observed in the dermis day 3 (d3) following TNT (Figures 1B and S1E). Imaging mass cytometry of the skin and WE tissue collected on d3 and d7, unlike other cell types present at the WE (Table S1and Figures 1B,C, S1F–K), showed that Exo_κ_ and Exo_mϕ_ were highly abundant in myeloid and epithelial cells, respectively (Figure 1D–I). Because d3 and d7 had a high abundance of Exo_mϕ_ in the epithelial cells, immunohistochemistry of RFP was performed on the d5 WE tissue. Exo_mϕ_ was found to be localized at the leading edge of the epithelial tongue (Figure 1J).

As background support for the significance of mϕ ↔ keratinocyte crosstalk in healing wounds of human subjects, we downloaded a single-cell RNA-seq data set from GEO (GSE165816). The data set includes 14 samples from 11 diabetic patients (two samples were collected from patients P3, P5, and P9; see Table S2). The cutaneous wounds from these 11 diabetic patients were monitored for 12 weeks and were divided into two subgroups: those whose ulcers healed and those whose ulcers did not heal. The wounds from 7 patients were identified as healing, while 4 patients had nonhealing cutaneous wounds (Table S2). The data set was reanalyzed using the Seurat v4.0.2 package in R. After applying quality control measures, 33,814 cells passed filtering and were normalized using the SCTransform method. The integration of these cells was performed through reciprocal principal component analysis (RPCA) in Seurat (Figure S2A). Following integration, an unsupervised principal component analysis (PCA) was carried out that identified nine distinct cell clusters (0–8). These clusters were then annotated based on the expression profiles of the top ten highly expressed marker genes, using well-established markers from PanglaoDB (Figure S2A). Notably, cluster 2 exhibited characteristic expression profiles of keratinocytes (KRT1^hi^, KRT10^hi^, and KRT14^hi^), while cluster 5 displayed features indicative of myeloid cells (LYZ^hi^ and CD45^hi^). Subsequent subcluster analysis at a resolution of 0.20 was performed on these two clusters, and the results were visualized in a UMAP plot (Figure S2B–D). Additionally, the CellChat package in R was utilized to construct a connectome, incorporating the total number of interactions and weighted interaction strength in each group (Figure S2E,F). The signaling pathways that were enriched in the healing group were the EGF and IGF signaling pathways (Figure S2G–J). CellChat identified a communication network between M1 ↔ K2 and M2 ↔ K1 subsets of mϕ and keratinocyte, respectively, in the healer that were compromised in nonhealers (Figure S2G–I). This line of evidence suggested that mϕ ↔ keratinocyte crosstalk is critical for successfully healing cutaneous wounds.

### Macrophage-Derived Exosomes Pack Mitochondrial Protein Cargo

**Figure 2:**
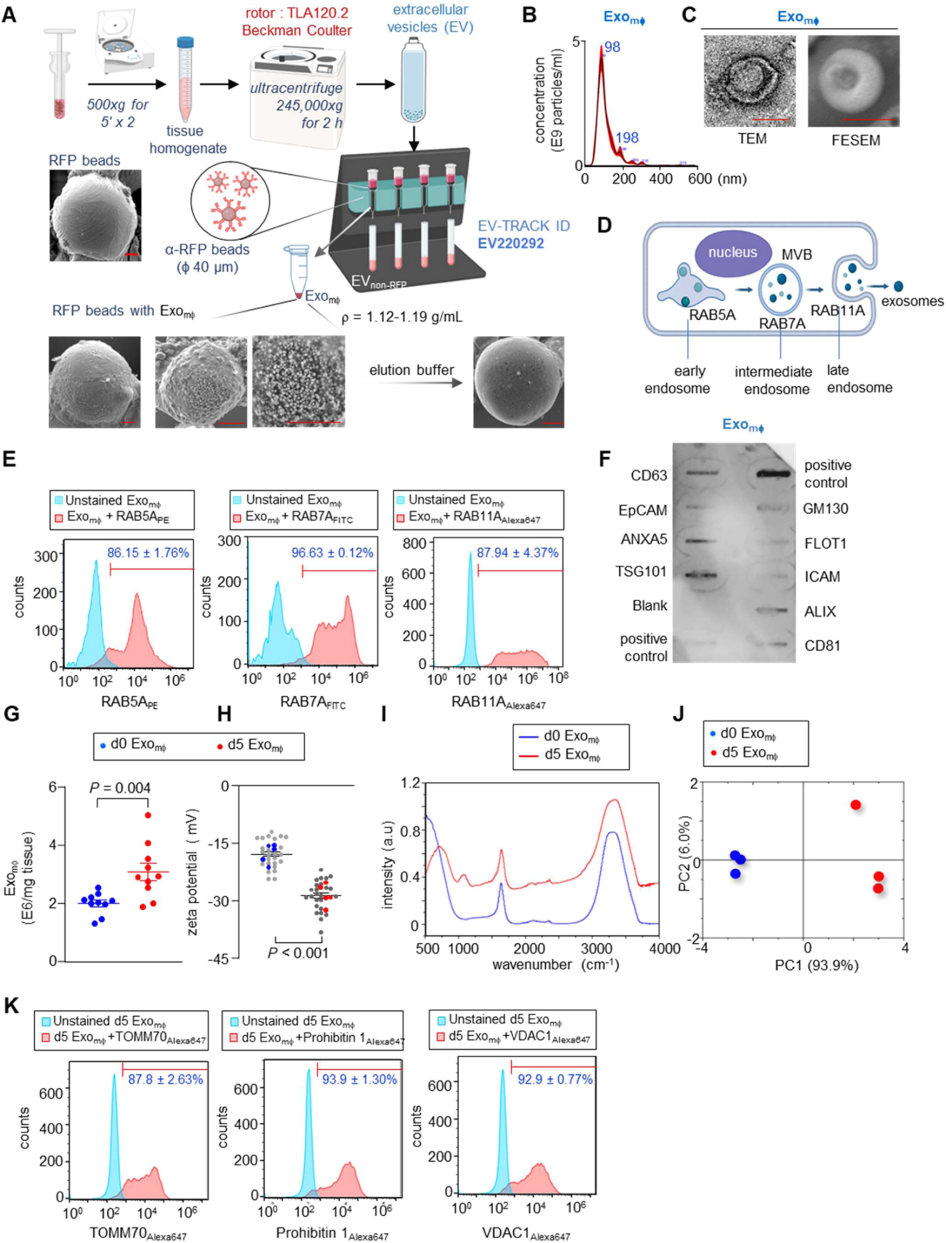
Macrophage-derived exosomes isolated from WE tissue showed an abundance of mitochondrial proteins. (A) Schematic diagram showing isolation of Exo_mϕ_ from murine WE tissue. Field emission scanning electron microscopy (FESEM) images demonstrate the presence of Exo_mϕ_ on RFP magnetic beads. Scale, 5 μm. (B) Particle size distribution of Exo_mϕ_ from murine WE tissue (*n* = 10). (C) Representative transmission electron microscopy (TEM) and FESEM images of Exo_mϕ_. Scale, 100 nm. (D) Schematic diagram of endosomal pathway showing early, intermediate, and late endosomal markers. (E) Bead flow cytometric analysis of murine Exo_mϕ_ conjugated with supermagnetic Dynabeads functionalized with the RFP antibody showing binding of RAB5A_PE_, RAB7A_FITC_, and RAB11A_AF647_ antibodies. The histogram demonstrates the shift in FITC and AF647 fluorescence after binding with the antibodies. The mean percentage of beads with Exo_mϕ_ was mentioned over the marker bar. (F) Antibody array of Exo_mϕ_ isolated from murine skin and WE tissue. ALIX, TSG101, CD63, CD81, FLOT1, exosomal marker; EpCAM = epithelial cell adhesion molecule; ANXA5, Annexin 5. *Positive control for HRP detection of exosomes derived from the human serum. (G) Exo_mϕ_ were isolated and quantified using NTA from murine skin and d5 WE tissue after nanotransfection with the *Lyz2* plasmid cocktail (*n* = 10). (H) Zeta potentials of Exo_mϕ_ isolated from murine skin and d5 WE tissue at physiological pH (pH 7.4). Each gray dot corresponds to one technical replicate, and the blue and red dots correspond to biological replicates (*n* = 5). (I) Fourier Transform Infrared (FTIR) spectra of Exo_mϕ_ isolated from murine skin and d5 WE tissue. (J) Two-dimensional (2D) score plot constructed from principal component analysis of FTIR spectra of Exo_mϕ_ isolated from murine skin and d5 WE tissue (*n* = 3). (K) Bead flow cytometric analysis of murine Exo_mϕ_ conjugated with supermagnetic Dynabeads functionalized with the RFP antibody showing binding of TOMM70_AF647_, Prohibitin 1_AF647_, and VDAC1_AF647_ antibodies. The histogram demonstrates the shift in fluorescence after binding with the antibodies. The mean percentage of beads with Exo_mϕ_ was mentioned over the marker bar. Data are representative of three independent experiments. Data in panels (D) and (E) are shown as mean ± standard error of the mean (SEM) and are analyzed by two-tailed unpaired Student’s *t*-test.

To study the significance of Exo_mϕ_ at the leading edge of the epithelial tongue, the Exo_mϕ_ was isolated from the WE tissue using differential ultracentrifugation and the immunomagnetic pull-down method, as previously reported by us.^19^ The RFP reporter protein was cloned “in-frame” with CD9, CD63, and CD81 driven by the *Lyz2* promoter. Thus, the pull-down of RFP^+^ exosomes using anti-RFP magnetic beads resulted in the separation of Exo_mϕ_ from the heterogeneous pool of EVs (Figure 2A). The Exo_mϕ_ was eluted from the beads using an elution buffer (Figure 2A). The size and concentration of Exo_mϕ_ were analyzed by nanoparticle tracking analysis (NTA) (Figure 2B). Transmission and field emission scanning electron microscopy of Exo_mϕ_ revealed the cup-shaped morphology (Figure 2C). The density of Exo_mϕ_ was found to be in the range of 1.12–1.19 g/mL. MISEV 2023 guidelines recommend that the purity of the isolated EVs is a critical determinant for downstream functional analysis.^30^ Although exosomes may share the same size and density as other membrane-originated nanosized ectosomes (Ø 50–1000 nm), there are some distinct biological differences. Unlike membrane-originated nanovesicles, endosomal-originated exosomes (Ø 50–150 nm) contain cargo with EXOmotif sequences that are selectively assorted and packaged for intercellular communications.^31−33^ Thus, along with tetraspanins, it is prudent to show the presence of endosomal markers (Figure 2D) in Exo_mϕ_.^34^ Incubation of the postadsorbed Exo_mϕ_ on RFP-trap bead’s surface (Figure 2E) with fluorescently tagged antibodies of endosomal markers followed by flow cytometry showed an increase in the fluorescence intensity (Figure 2E). The presence of other exosomal markers in abundance such as ALIX, TSG101, ANXA5, Caveolin-1, and LAMP1 (Figures 2F and S3A) with the negligible presence of histone proteins (Figure S3B) demonstrated the purity of the isolated Exo_mϕ_ from the tissue homogenate.

The presence of Exo_mϕ_ was found to be significantly higher at d5 WE tissue (Figure 2G). Unlike the d0 Exo_mϕ_, the zeta potential (ζ) of d5 Exo_mϕ_ was significantly lower, suggesting the possibility of faster uptake by other recipient cell types (Figure 2H). Raman and Fourier Transform Infrared (FTIR) spectroscopic analyses of Exo_mϕ_ isolated from the skin (d0) and d5 WE tissue demonstrated spectral differences across the proteins, lipids, and nucleic acid domains (Figures 2I, S3C and Tables S3, S4). The dimensionality of the data set was reduced by transforming the original variables into principal components (PC). This multivariate statistical technique was applied to FTIR spectroscopy data, facilitated by the PCA module within the OriginPro software suite. The analytical focus was centered on delineating spectral variance comparing d0 and d5 Exo_mϕ_ samples. The results were visually represented in a 2D score plot. This separation was predominantly attributed to the first principal component (PC1), which accounted for 93.9% of the total spectral variance, in stark contrast to the second principal component (PC2), which represented only 6.0% of the variance (Figure 2J). Additionally, PCA applied to Raman spectroscopic data revealed a similar trend, albeit with different variance proportions: PC1 constituted 56.2% of the total spectral variance, while PC2 accounted for 17.3% (Figure S3D).

Mitochondrial protein screening of d5 Exo_mϕ_ identified a few distinct proteins that were absent in the injured skin (d0). Interestingly, mitochondrial proteins such as the translocase of outer membrane protein 70 (TOMM70), which was not detected in d0 Exo_mϕ_ (Figure S3E), along with other mitochondrial proteins such as Prohibitin 1 and VDAC1, were found in high abundance in d5 Exo_mϕ_ (Figure 2K). Furthermore, the scRNA-seq data set downloaded from the publicly available GEO database (GSE165816) of diabetic patients with healing and nonhealing wounds also identified a high abundance of TOMM70 in the K2 keratinocyte subset in the healing group (Figure S2K). Transfer of mitochondrial cargo from airway mϕ to T-cells has been shown to regulate their bioenergetics.^35^ Prior *in vitro* research also acknowledges the presence of mitochondrial components in the secretome of cell-culture-conditioned media.^36−40^ In recent years, increasing evidence of mitochondrial cargo shuttled in extracellular vesicles (EV), suggesting that EV could be used as vectors for mitochondrial transfer.^35,38,41,42^ In light of these evidences, our findings suggest that there exists a mechanism for the selective packaging of mitochondrial proteins in exosomes, unlike the abundance of specific mitochondrial proteins in membrane-originated EV.^34^

Studies investigating EV carrying mitochondrial components are inherently complex due to two primary factors. First, EV encompasses numerous families of heterogeneous vesicles differing in size, density, and origin.^43−45^ Concerns with inappropriate separation techniques of multiple possible identities of similar size vesicles limit the ability to interpret data and delineate specific mechanistic underpinnings.^46−49^ Exosomes, unlike other membrane-originated particles, carry a specific assortment of cargo that are selectively enclosed within these vesicles of endocytic origins.^50,51^ This process of cellular communication, characterized by interactions facilitated through specific membrane vesicles, stands apart from other mechanisms involving the shedding of membrane vesicles that may also carry biomolecules but lack selective packaging.^52,53^ Second, given the size of mitochondria, it is plausible that following either mitophagy or fission, a part is expunged from the cells as byproducts in a metabolic recycling pathway rather than an intercellular signaling pathway.^54^ For example, it is been reported that PTEN-induced putative kinase 1 (PINK1), a protein involved in mitophagy, drives the production of EV with mitochondrial cargo.^55^ Contradicting this idea, there are reports that cells have a distinct mechanism to sort the cargo that is dependent on selective targeting to one of two distinct mitochondria-derived vesicular pathways.^56^

**Figure 3:**
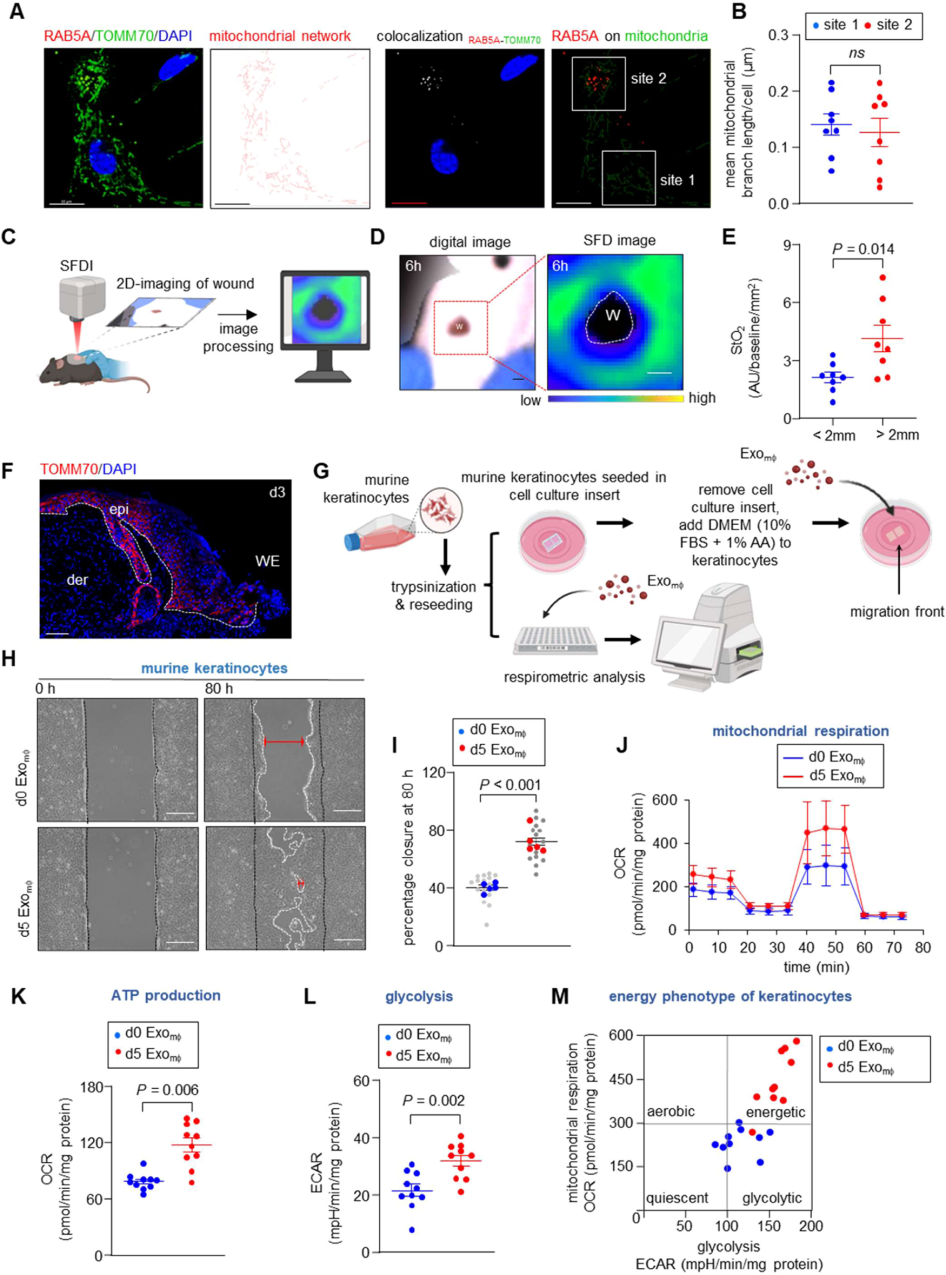
TOMM70 is actively packaged in Exo_mϕ_ that augments keratinocyte migration. (A) Representative immunocytochemistry of RAB5A (red) and TOMM70 (green) in murine proinflammatory mϕ. TOMM70 signal was used for mitochondria morphology analysis. MiNA tool on the ImageJ interface was used to show the skeletonized images of the mitochondrial network. Colocalization of TOMM70 with RAB5A was shown as white dots. The red fluorescence of RAB5A was superimposed with the mitochondrial network (green) for the quantification of mean mitochondrial branch length. Scale, 10 μm. (B) Quantification of the mean mitochondrial branch length from areas with and without RAB5A colocalization (*n* = 8). (C) Schematic diagram demonstrating the experimental setup of Spatial Frequency Domain Imaging (SFDI) of the murine dorsal excisional wound. (D) Digital and SFDI photographs of murine dorsum 6 h post wounding. Scale, 1.5 mm (E). (E) Quantification of cutaneous tissue O_2_ saturation at WE (<2 mm) and skin (>2 mm) analyzed from SFD images (*n* = 8). (F) Immunohistochemistry of TOMM70 with DAPI counterstaining in d3 murine WE tissue. White dashed lines indicate the dermal–epidermal junction. Scale, 100 μm. (G) Schematic experimental design for respirometric study and cell migration assay. (H) Representative phase-contrast microscopic images of murine keratinocytes at 0 h and 80 h showing migration following treatment with Exo_mϕ_ isolated from murine skin (d0) and d5 WE tissue. Scale 200 μm. (I) Keratinocyte migrations were quantified and expressed as percentage closure. For each image, the distance of the migrating front at the top, middle, and bottom was measured (gray dots) and the mean value was plotted (blue, red) (*n* = 5). (J) Baseline OCR in murine keratinocytes after the addition of Exo_mϕ_ isolated from murine skin (d0, blue dots) and d5 WE tissue (red dots) was plotted graphically. (K, L) ATP production and glycolysis in murine keratinocytes as calculated from OCR and ECAR (*n* = 10). (M) Energy phenotype of keratinocytes analysis as calculated from OCR and ECAR (*n* = 10). Data in panels (B, E, I, K, and L) are shown as mean ± SEM and are analyzed by two-tailed unpaired Student’s *t-*test.

Intrigued by our findings, we posed two fundamental questions. First, we need to determine whether our method isolates mitovesicles or not. Mitovesicles lack exosomal protein markers and several biosynthetic and import proteins.^42^ Furthermore, proteins such as TOMM involved in cytosol-to-mitochondria peptide import machinery are scarcely found in mitovesicles.^42^ Our findings to demonstrate the presence of endosomal markers along with the presence of TOMM70 led to the conclusion that the isolated d5 Exo_mϕ_ are distinct from mitovesicles. Second, we asked whether these endosomal-originated Exo_mϕ_ are amphisomes containing mitochondrial fragments.^57^ Amphisomes are formed by the fusion of endosomes with selective sequestration of mitochondrial fragments by autophagosomes within cells.^58^ TOMM70 is a versatile adaptor protein anchored in the outer membrane of mitochondria (Figure S4A).^59^ Mitochondrial network analysis of proinflammatory mϕ performed following immunostaining with TOMM70 and RAB5A, a marker of early endosomes, showed colocalization in the cytosol without any mitochondrial fragments (Figure 3A). No significant difference in the mitochondrial branch length was observed between places with and without RAB5A colocalization (Figure 3B). Furthermore, the colocalization of TOMM70 with DRP1, a protein responsible for cleaving only the outer mitochondrial membrane, and substantially less colocalization with OPA1, a protein involved in mitophagy and responsible for cleaving both outer and inner mitochondrial membranes,^60,61^ lead to the notion that packaging of TOMM70 within the endosomal-originated exosomes in proinflammatory mϕ may be an active process (Figure S4B).

### Epidermal TOMM70 is Hypoxia-Sensitive and Required for Keratinocyte Migration

Export of TOMM70 from blood-borne ωmϕ via exosomes and uptake by leading-edge keratinocytes question the fate of epidermal TOMM70 in the context of mϕ-keratinocyte crosstalk. Spatial Frequency Domain Imaging of murine dorsal excisional wound at 6 h demonstrated that the WE (<2 mm) were significantly hypoxic (Figure 3C–E). The abundance of TOMM70 was found to be low at the WE epidermis (Figure 3F). Compared to the normoxic environment, murine keratinocytes when subjected to experimental hypoxia for 12 h showed a significantly low abundance of TOMM70 suggesting that TOMM70 is vulnerable to hypoxia (Figure S4C,D). Interestingly, the mitochondrial membrane potential did not change significantly at 12 h (Figure S4E). This observation warrants further in-depth mechanistic insight into the role of TOMM70. Based on the available literature, we posit that this 70 kDa adaptor protein is anchored in the mitochondrial outer membrane and plays a critical role in maintaining mitochondrial function and quality.^59^ TOMM70 selectively recognizes and binds to precursor proteins in the cytoplasm that are destined for the mitochondria. Furthermore, its tetratricopeptide repeat (TPR) domain also facilitates the import of preproteins lacking a positively charged mitochondrial targeted sequence.^59,62^ For example, TOMM70 directs the N-terminal mitochondrial targeting sequence (MTS) of serine/threonine PINK1 into the translocation pore formed by TOMM40.^63^ TOMM40 translocates PINK1 to the inner mitochondrial membrane (IMM) where it was sequentially cleaved by the presenilin-associated rhomboid-like (PARL) mitochondrial processing peptidase and degraded through the N-end rule pathway.^64−67^ In damaged mitochondria, transport of PINK MTS was compromised, resulting in the stabilization of PINK on the outer mitochondrial surface and initiating mitophagy with PARKIN.^36,68^ It is plausible that the loss of TOMM70 from a healthy mitochondrion may also initiate mitophagy without a significant drop in the mitochondrial membrane potential. At the leading WE keratinocytes, such loss of otherwise functional mitochondria is detrimental to the reepithelialization process that relies on mitochondrial bioenergetics for keratinocyte proliferation and migration. This was further corroborated by the observation that knocking down TOMM70 results in impaired keratinocyte migration (Figure S4F,G). Furthermore, the addition of Exo_mϕ_ to hypoxic keratinocytes resulted in the fusion with mitochondria (Figure S4H). Thus, we posit that the addition of Exo_mϕ_ to keratinocytes should increase keratinocyte migration along with an increase in mitochondria bioenergetics (Figure 3G). Compared with d0 Exo_mϕ_, the addition of d5 Exo_mϕ_ showed a significant increase in keratinocyte migration (Figure 3H–I). The abundance of TOMM70 was significantly higher in keratinocytes exposed to d5 Exo_mϕ_ (Figure S4I,J). Furthermore, d5 Exo_mϕ_ resulted in increased mitochondrial bioenergetics via the glycolytic pathway (Figures 3J–M and S4K,L). However, no significant difference in keratinocyte proliferation was observed (Figure S4M,N).

### TOMM70 Packaging in Exo_mϕ_ is Responsive to Exosomal Uptake by WE Keratinocytes

**Figure 4:**
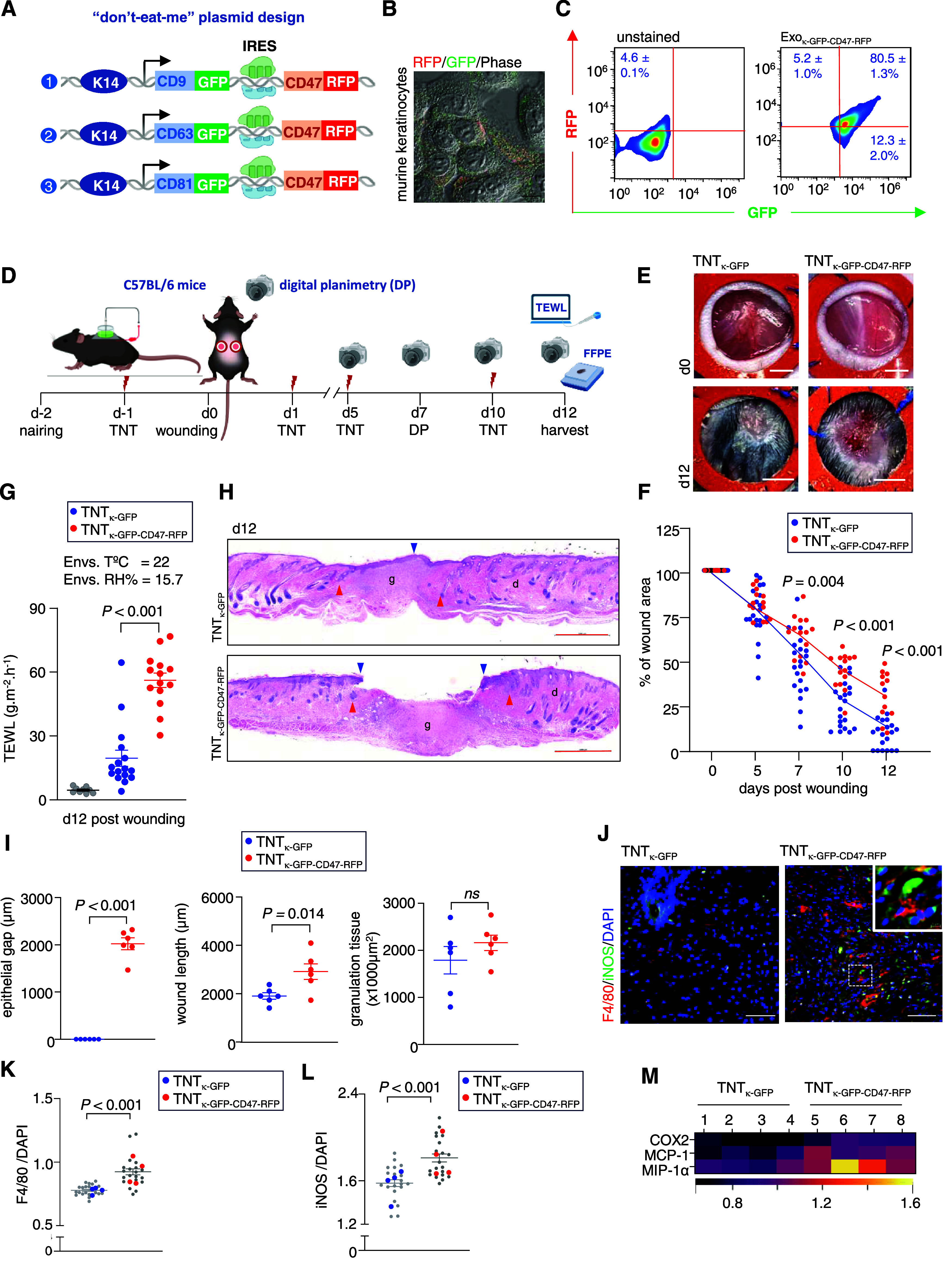
Bidirectional crosstalk between WE mϕ and resident keratinocytes is critical for functional wound closure. (A) Design of *Krt14*-promoter-driven tetraspanins plasmid-connected via the IRES element with “don’t eat me”-CD47 sequence with “in-frame” GFP and RFP reporter. (B) Representative phase-contrast confocal images showing coexpression of RFP (red) and GFP (green) in murine keratinocytes. Scale, 20 μm. (C) Flow cytometric analysis of murine Exo_κ_ on pan CD magnetic beads showing dual positivity of GFP and RFP on “don’t eat me” Exo_κ-GFP-CD47-RFP_. (D) Schematic experimental design. (E) Digital photographs of the excisional stented punch wound (6 mm) at d0 and d12 post wounding in C57BL/6 mice treated with either TNT_κ-GFP_ or TNT_κ-GFP-CD47-RFP_. (F) Quantification of the wound area by digital planimetry following TNT_κ-GFP_ or TNT_κ-GFP-CD47-RFP_. Scale, 2 mm (*n* = 20). (G) TEWL in C57BL/6 mice at d12 post wounding following TNT_κ-GFP_ or TNT_κ-GFP-CD47-RFP_. The gray dots represent normal skin (*n* = 16). (H) Representative H&E staining of wounds of C57BL/6 mice at d12 post wounding following either TNT_κ-GFP_ or TNT_κ-GFP-CD47-RFP_. Scale, 1000 μm. The blue vertical arrowhead represents complete reepithelialization. The red arrowhead represents the WE. (I) Morphometric analysis showing an epithelial gap, wound length, and granulation tissue area in C57BL/6 mice at d12 post wounding following either TNT_κ-GFP_ or TNT_κ-GFP-CD47-RFP_ (*n* = 6). (J) Representative coimmunofluorescence staining of F4/80 (red) with iNOS (green) and DAPI counterstaining in the WE granulation tissue at d12 post wounding in C57BL/6 mice following either TNT_κ-GFP_ or TNT_κ-GFP-CD47-RFP_. (K, L) Quantification of F4/80 and iNOS intensity in WE tissue at d12 post wounding in C57BL/6 mice following either TNT_κ-GFP_ or TNT_κ-GFP-CD47-RFP_. Each corresponds to one quantified ROI, except the blue and red dots, which correspond to the mean of each mouse. At least 5 ROI per mouse (*n* = 4 and 5). (M) Heat map showing the expression of proinflammatory markers COX2, MCP-1, and MIP-1α in WE granulation tissue at d12 post wounding in C57BL/6 mice following either TNT_κ-GFP_ or TNT_κ-GFP-CD47-RFP_ (see Figure S5h). Data in panels (C, F, G, I, K, and L) are shown as mean ± SEM and are analyzed by two-tailed unpaired Student’s *t test*.

Given the importance of proinflammatory Exo_mϕ_ in keratinocyte migration, we asked why keratinocyte migration is compromised in diabetes, given the presence of profound proinflammatory mϕ. Our previous work on human chronic wound fluid has demonstrated that the uptake of keratinocyte-derived exosome (Exo_κ_) by ωmϕ is compromised under diabetic conditions.^20^ We hypothesize that the TOMM70 packaged in WE Exo_mϕ_ may be directed by cues from keratinocytes. To test this hypothesis, “don’t eat me” Exo_κ_ was constructed such that the *Krt14*-promoter-driven tetraspanin plasmids with the GFP reporter were connected via the IRES element with “don’t eat me”-CD47 sequence with the in-frame RFP reporter (Figure 4A). Superresolution confocal microscopy demonstrated the expression of both tetraspanins (green) and CD47 (red) in murine keratinocytes (Figures 4B and S5A). The Exo_κ_ isolated from the conditioned media of these transfected murine keratinocytes showed the presence of both GFP and RFP (Figures 4B,C and S5B). Unlike Exo_κ_, the uptake of Exo_κ-GFP-CD47-RFP_ (Exo_κ_ with a “don’t eat me” signal) was significantly compromised by the proinflammatory mϕ (Figure S5C,D). To test these findings in cutaneous wounds, either the *Krt14*-promoter-driven tetraspanin reporter plasmids or the same plasmids with the CD47 sequence (“don’t eat me” signal) were delivered on murine dorsal skin (Figure 4D). Unlike the mice where keratinocyte ↔ ωmϕ crosstalk is not compromised, digital planimetry demonstrated compromised wound closure at d12 post wounding in the TNT_κ-GFP-CD47-RFP_ group (Figure 4E,F). Furthermore, functional wound closure as measured by transepidermal water loss (TEWL) was also significantly higher in the TNT_κ-GFP-CD47-RFP_ group (Figure 4G). Analytical histology demonstrated that although there is no significant difference in the wound length or granulation tissue area, the TNT_κ-GFP-CD47-RFP_ group showed compromised reepithelialization, suggesting that uptake of Exo_κ_ by proinflammatory mϕ is critical for keratinocyte migration (Figures 4H,I and S5E). Furthermore, no significant difference in neutrophil abundance was observed (Figure S5F,G), and the TNT_κ-GFP-CD47-RFP_ group demonstrated a high abundance of mϕ at d12 with increased expression of proinflammatory markers such as iNOS, COX2, MCP-1, and MIP-1α (Figures 4J–M and S5H,I). This condition mimics what is observed during diabetes, such as the compromised uptake of Exo_κ_ by proinflammatory mϕ,^20^ persistence of proinflammatory mϕ,^69−72^ and stalled keratinocyte migration.^73,74^ Because of the stalled reepithelialization, the expression of junctional proteins and other differentiation markers were compromised in the TNT_κ-GFP-CD47-RFP_ group, thereby further supporting high TEWL in this group (Figures S6 and S7).

The metabolic cost of cell migration, while substantial, remains underexplored in quantitative detail. A 2-phase model of the cell cytoplasm asserts that the effectiveness of cell migration, in terms of energy and mechanics heavily relies on the physical properties of the microenvironment.^75^ Actin polymerization-driven migration is considered inefficient in high hydraulic resistance conditions. Theoretical and practical evidence suggests that the cost of migration is affected by a combination of matrix flexibility and the level of restriction; higher cell stiffnesses and matrix stiffnesses, along with increased confinement result in higher energy demands for migration activities.^76^ These factors limit migration abilities and lead cells to move toward areas of least confinement to minimize energy expenditure.^76^ However, at the WE, cells migrate with minimal physical resistance, facilitating the subsequent proliferation of cells in trailing positions. Normally, migration precedes proliferation as cells relocate before initiating rapid division.^77,78^ Additional studies have shown that glycolysis, associated with cytoskeletal activity, enhances the migration speed by enabling rapid adaptation to fluctuating energy demands at the membrane level, often coupled with increased glucose uptake during heightened energy expenditure.^79,80^ Although glycolysis generates ATP up to 100 times faster than oxidative phosphorylation (OXPHOS), it offers a lower energy yield, creating a trade-off between the production rate and efficiency.^81^ Cells may rely on glycolysis for immediate energy requirements and utilize mitochondrial rearrangement for precise subcellular energy distribution.^81,82^ This bidirectional crosstalk between migration and proliferation may act as a compensatory mechanism to initiate the healing process, even under adverse hypoxic conditions.

Thus, in this study, we have demonstrated that following injury, the Exo_mϕ_ was localized at the leading-edge keratinocytes. The Exo_mϕ_ were abundant in TOMM70 and were delivered to the leading-edge keratinocytes to compensate for the low abundance of TOMM70 (Figure S8A–C). Such TOMM70-enriched Exo_mϕ_ fuses with the mitochondria in the leading-edge keratinocytes and replenishes the TOMM70 that was lost due to hypoxia. Such restoration of mitochondrial bioenergetics via the glycolytic process provided the necessary energy to support keratinocyte migration. Selective disruption of exosomal communication between WE keratinocytes and Exo_mϕ_ resulted in a notable delay in reepithelialization and impeded functional wound closure. Notably, this observation underscores the significant impact of such intercellular communication on wound tissue bioenergetics, which is crucial for meeting the metabolic demands associated with the closure of wounds. Taken together, our findings indicate that keratinocyte ↔ ωmϕ ↔ keratinocyte crosstalk via exosomes is critical for functional wound closure.

## Conclusions

In summary, we identified that selective interruption of exosomal crosstalk between ωmϕ and WE keratinocytes significantly delayed reepithelialization and impaired functional wound closure. These findings collectively suggest that not only Exo_mϕ_ but bidirectional exosomal crosstalk between ωmϕ and WE keratinocytes is of utmost importance for functional wound healing. Although our study elucidates the critical aspects of keratinocyte ↔ ωmϕ ↔ keratinocyte crosstalk via the exosome in functional wound closure, several avenues remain unexplored and warrant further investigation for an in-depth understanding of this bidirectional paracrine signaling. First, the observation that compromising exosomal uptake by ωmϕ and not just inhibiting exosomal cargo packaging compromises keratinocyte migration, suggesting that the surface composition of Exo_κ_ is a critical factor supporting exosomal packaging in ωmϕ. An in-depth surface characterization of Exo_κ_ is thus warranted. Second, how the Exo_mϕ_ cargo changes following the uptake of Exo_κ_ must be investigated. Finally, this work demonstrates that Exo_mϕ_ fuses with the mitochondria of the keratinocytes. It will be critical to understand whether Exo_mϕ_ selectively fuses with low-potential mitochondria. Finally, how TOMM70 achieves bioenergetic functionality in the keratinocytes remains to be addressed.

## Methods

### Cells and Cell Culture

Murine keratinocytes (Kera308; # 400429) obtained from CLS Cell Lines Service GmbH were cultured in Dulbecco’s modified Eagle’s medium (DMEM) supplemented with 4.5 g/L glucose, 2 mM glutamine, and 10% fetal bovine serum.^83^ Mouse macrophage cell line (RAW 264.7) was purchased from ATCC (RAW 264.7; # TIB-71) and was cultured in Dulbecco’s high-glucose (4.5 g/L) modified Eagle’s medium (Life Technologies, Gaithersburg, MD). Murine ωmϕ was isolated by subcutaneously implanting sterile circular poly(vinyl alcohol) (PVA) sponges (8 mm) on the backs of 8–12-week-old mice.^71^ CD11b^+^ sponge-infiltrated ωmϕ were isolated from the d3 wound infiltrate using the CD11b magnetic microbead-based sorting approach described previously.^71^ The isolated murine ωmϕ was cultured in RPMI-1640 media. Murine brain endothelial cells were purchased from ATCC (bEnd.3; # CRL-2299) and were cultured in Dulbecco’s high-glucose (4.5 g/L) modified Eagle’s medium (Life Technologies, Gaithersburg, MD). Primary mouse embryonic fibroblasts were purchased from Millipore Sigma (# PMEF-HL-C) and were cultured in DMEM medium supplemented with 10% fetal bovine serum, 100 μg/mL streptomycin, 100 U/mL penicillin, 0.25 μg/mL amphotericin, and 1× MEM nonessential amino acids (all from Thermo Fisher Scientific). All cells were maintained in a conventional culture incubator with 5% CO_2_ humidified air. Exosome-depleted FBS (Gibco, no. A2720803) was used in all experiments that involved the isolation or uptake of exosomes.

### Mouse Model

Male C57BL/6 mice (aged 8–10 weeks) were obtained from Jackson Laboratory. All animal studies were performed in accordance with protocols approved by the Laboratory Animal Resource Center (LARC) of Indiana University and the Division of Laboratory Animal Resources (DLAR) at the University of Pittsburgh. The animals were ear-tagged and grouped randomly using a computer-based algorithm (www.random.org).

Two full-thickness excisional wounds of 6 mm diameter, equidistant from the midline, were created on the dorsal skin of mice with a 6 mm disposable biopsy punch.^84^ The excisional wounds were splinted with a 0.5 mm silicon sheet (Grace Bio-Laboratories; # 664581) to prevent contraction, thereby allowing wounds to heal through granulation and reepithelialization.^19^ For isolation of exosomes from the WE, four 8-mm-diameter full-thickness excisional wounds were developed on the dorsal skin of mice with an 8 mm disposable biopsy punch without any stent.^29^ Mice were anesthetized with low-dose isoflurane (1.5–2%) inhalation per standard recommendation. Each wound was digitally photographed at the time point indicated. The macroscopic wound area was calculated as a percentage of the wound area immediately postsurgery by processing photographs taken at various time points using ImageJ and was calculated as the percentage of closure. The animals were euthanized at the indicated time, and WE samples were collected for analysis. For the WE harvest, 1–1.5 mm of tissue from the leading edge of the wounded skin was excised around the entire wound. Tissue from the wounds (skin) was snap-frozen and stored at −80 °C until cell-specific exosomes were isolated, as described above. Wound-edge tissues were collected either in 4% paraformaldehyde or in an optimal cutting temperature (OCT) compound for immunohistochemistry.^85,86^

### Exosome Isolation from Cell Culture Media

Exosomes were isolated from cell culture-conditioned media supplemented with 10% Exosome-Depleted FBS (Thermo Fisher Scientific; Gibco, # A2720803) as described previously.^19^ Briefly, the conditioned media was centrifuged at 10,000 *g* for 45 min, and the supernatant was collected. Differential ultracentrifugation (Beckman Coulter Optima Max-XP Ultracentrifuge, rotor TLA120.2) was performed to isolate exosomes from the supernatant followed by immunomagnetic separation using CD Dynabeads as described previously.^20^ The exosomes were not eluted from magnetic beads for flow cytometry analysis. For nanoparticle tracking analysis (NTA), transmission electron microscopy (TEM), and field emission scanning electron microscopy (FESEM) imaging, exosomes were eluted from the beads using an elution buffer (ExoFlow Exosome Elution Buffer, System Biosciences; # EXOFLOWBUFR-2). This method was reported in the EV track (EV-TRACK ID: EV190103) and received an EV metric score of 100%.

### Isolation of Murine Macrophage-Derived Exosomes from WE Tissue

Macrophage-derived exosomes (Exo_mϕ_) were isolated from murine WE tissue following tissue nanotransfection with a cocktail of *Lyz2* promoter-driven plasmids encoding murine CD63, CD9, and CD81 with an “in-frame” RFP reporter. The murine skin and WE tissue were collected and homogenized, before being suspended in phosphate-buffered saline (PBS) and vortexed to release exosomes from the tissue. The exosome suspension was briefly centrifuged, and the supernatant was collected. The collected supernatant was centrifuged at 5000 *g* for 15 min followed by 10,000 *g* for 45 min at 4 °C. The supernatant was incubated overnight at 4 °C with RFP-Trap magnetic agarose beads (Chromotek; # rtma-100) (12 μL RPF-Trap beads per 0.15 g tissue). RFP-Trap magnetic agarose beads were prewashed with a dilution buffer (10 mM Tris/Cl pH 7.5, 150 mM NaCl, 0.5 mM EDTA (adjust pH at 4 °C)) and blocked with 3% bovine serum albumin (BSA) before adding to the supernatant to avoid nonspecific binding. The RFP-trap beads were magnetically separated the next day to remove the eluent and washed thrice with the dilution buffer. Exosomes were eluted from intact beads by using an elution buffer, and Exo_mϕ_ pellets were collected by ultracentrifugation (1.5 h at 245,000 *g*). This method was submitted to EV track (EV-TRACK ID: EV220292) and received a preliminary score of 100%.

### Density of Exo_mϕ_

The density of isolated Exo_mϕ_ was analyzed by loading the exosomal dispersion on top of a discontinuous sucrose step-gradient ranging from 0.5–2.5 M sucrose (Sigma-Aldrich; # S5-500) as described previously.^20^ The density of exosomes was calculated to be in the range of 1.12–1.18 g/mL.

### Flow Cytometry

The unconjugated antibodies were labeled with CF Dyes using Mix-n-Stain Antibody Labeling Kits (Biotium; # 92273, # 92275, # 92279) or by Alexa Fluor Antibody Labeling Kits (Abcam; # A20186) as per manufacturer’s instructions before use. Exosome markers were assessed using PE anti-RAB5A (Abcam; # ab302987; 1:200), FITC anti-RAB7A (Abcam; # ab137029; 1:200), Alexa Fluor 647 anti-RAB11A (Invitrogen; # 71-5300; 1:200), FITC anti-Caveolin-1 (Novus Biologicals; # NB100-615AF488; 1:200), and FITC anti-LAMP1 (Abcam; # ab24871; 1:200) by incubating the antibodies at the noted concentrations for 60 min at room temperature with the exosome-conjugated RPF-Trap beads. Other markers were also accessed using Alexa Fluor 647 anti-TOMM70 (Abcam; # ab289977; 1:250), Alexa Fluor 647 anti-Prohibitin 1 (Novus Biologicals; # NBP2-67334; 1:250), Alexa Fluor 647 anti-VDAC1+VDAC3 (Abcam; # ab14734; 1:100), PE anti-RFP (Rockland Immunochemicals; # 600-901-379; 1:200), and FITC anti-GFP (Abcam; # ab6556; 1:150) as described previously.^20,86,87^ An Accuri C6 flow cytometer (Accuri Cytometers, MI) was used to analyze the samples. Data from 5000–10,000 events were collected at a flow rate of 250–300 events/s and analyzed using FlowJo software (Tree Star, OR). FITC fluorescence and PE fluorescence were determined using the FITC channel and PE channel, respectively.

### Nanoparticle Tracking Analysis

The average size and concentration of Exo_mϕ_ were measured using an NTA (Nanosight NS300) equipped with an SCMOS camera (Malvern, Worcestershire, U.K.) and a 532 nm laser. All measurements were conducted at a sample dilution of 1:100–1:1000. Prior to measurement, the instrument was calibrated using 100 nm standard latex spheres (dilution 1:1000) in Milli-Q. Data were analyzed by NTA 3.0 software (Malvern Instruments) as described previously.^19,20^

### Zeta Potential Analysis

The surface charge (zeta potential) measurement of Exo_mϕ_ was determined by diluting the exosome suspension in double-distilled water (1:20) using Zetasizer (Nano-Z, Malvern Instruments Ltd., U.K.) as described previously.^20,88^ The samples were tested in a volume-weighted size distribution mode.

### Electron Microscopy

Transmission electron microscopy (TEM) measurements were performed on a carbon-coated copper grid (300-μm mesh) that was subjected to a glow discharge treatment prior to sample loading. An exosome sample was spotted over the entire grid area. Excess solvent was removed by blotting with a filter paper after ∼30 s. The grids were stained for 30 s with 2% uranyl acetate, dried, and imaged on a JEOL JEM 1400plus transmission electron microscope equipped with a 4000 × 4000-pixel Gatan CCD camera.

The morphologies of Exo_mϕ_, RFP-trap beads surface, RFP-trap beads surface after exosome binding, and RFP-trap beads surface after elution of exosomes were accessed by a field emission scanning electron microscope (JEOL 7800F, JEOL Japan). The exosome pellet after ultracentrifugation was redispersed in 10% glutaraldehyde for 10 min at room temperature (RT). Fixed Exo_mϕ_ was drop-cast onto the stubs containing glass coverslips mounted on a carbon tape and dried in a vacuum chamber for at least 12 h before analysis. RFP-trap bead samples (without exosomes) were directly dropped onto the stubs containing a carbon tape. Samples were imaged after gold sputter coating at a beam energy of 10 kV.^20^

### Raman Spectroscopy

Exo_mϕ_ was characterized by Raman spectroscopy using a Raman microscope (Horiba Xplora Plus) as described previously.^20^

### Fourier Transform Infrared Spectroscopy

Fourier transform infrared (FTIR) spectra of Exo_mϕ_ were recorded using a Nicolet iS10 FTIR spectrometer operating in transmission mode using the ATR method in the range of 4000–400 cm^–1^. From each sample, a drop of 2 μL was placed onto the diamond, and the spectrum was recorded for each sample. All spectra were recorded from five different biological replicates in three technical replicates averaged. For the control experiment, PBS and serum-free DMEM measurements were done in triplicate. All spectra were plotted using OriginPro 8.0 software.

### Single-Cell RNA Sequencing Analysis

Single-cell RNA-seq data set of 14 samples from 11 diabetic patients with healing (*n* = 7) and nonhealing (*n* = 4) diabetic foot ulcer (DFU), publicly available with accession number GSE165816 that we utilized previously,^84^ was reanalyzed using Seurat v4.0.25 package in R.^89^ Briefly, the processed data set was downloaded, examined for read quality in R, and stored as Seurat objects. Samples were quality-filtered using the “subset” module for multiple parameters including mitochondrial content < 15%, cells expressing >200 genes, and genes uniquely expressed in >3 cells and set for downstream analysis. Quality filtered data of 33,814 cells were normalized using the SCTransform^90^ and integrated using the reciprocal principal component analysis (RPCA) function in Seurat. Further, unsupervised analysis of the integrated samples was performed using the principal component analysis (PCA). Principal components thus obtained were projected to identify the cluster of cells using “FindNeighbors” followed by “RunUMAP” (Uniform Manifold Approximation and Projection) and “FindClusters” (at 20% resolution) analyses in Seurat. Nine clusters of cells (0–8) were identified and subsequently annotated to different cell types based on the established marker genes documented in PanglaoDB.^84,91^ The expression profile of compartment-specific signature genes was visualized by using the “VlnPlot” function of Seurat.

Clusters 2 (KRT1^hi^, KRT10^hi^, KRT14^hi^) and 5 (LYZ^hi^, CD52^hi^) consisting of 3010 and 4366 cells, respectively, were isolated using the “subset” module in Seurat. These cells were reclustered using unsupervised PCA analysis as described previously. The obtained subsets were projected in a UMAP plot and labeled for originally isolated compartments based on the expression of respective signature genes.^84,89^ In addition, the top five marker genes of each subset were obtained using Seurat’s “FindAllMarkers” function, and their relative expression across the subsets was illustrated in a dot plot.^84,89,91^

To investigate the cell communication among these subclusters (keratinocytes and myeloid cells) in DFU-healing and nonhealing groups, CellChat was employed.^92^ The connectome thus established among the subclusters was visualized as circle-net plots using the “netVisual_circle” module of CellChat and compared between the groups. These connectomes were further explored for significantly enriched signaling pathways ranked based on relative information flow (i.e., cumulative interaction probability among all pairs of cell types) computed using the “rankNet” function of CellChat. To identify the crucial subsets actively involved in their connectome, the expression profile of key genes, as noted in Figure S2H–J, was extracted and visualized using the “VlnPlot” function of Seurat. Selective signaling pathways supporting the hypothesis were investigated in the context of their connectome with other subtypes and discussed, along with the expression profile of the participating molecules.

### Exosome Antibody Array

Exosome markers in Exo_mϕ_ were analyzed using the Exo-Check Exosome Antibody Array kit (System Biosciences; # EXORAY210B-8) as per the manufacturer’s protocol. Briefly, 50 μg of protein was used for the array and blots were developed and imaged under Azure biosystems.^20^ Protein concentration in Exo_mϕ_ was determined by a bicinchoninic acid (BCA) assay.

### Exosome Uptake Assay

Keratinocyte-derived exosomes with and without the “don’t eat me” (*Krt14*-promoter-driven tetraspanins connected via the IRES element with “don’t eat me”-CD47 sequence) signals were isolated from cell-culture-conditioned media of transfected murine keratinocytes. For cellular uptake of these exosomes by mϕ, exosome concentration was measured by NTA and labeled with ExoGlow-Membrane EV Labeling Kit (System Biosciences; # EXOGM600A-1). The ExoGlow-labeled exosomes (10^8^ particles) were added to the d3 murine mϕ and live-cell imaging was performed using an LSM 880 confocal microscope (Zeiss) as described previously.^20^

### *In Vitro* Transfection

Lipofectamine 3000 Transfection Reagent (Invitrogen; # L3000015) was used to transfect the cells with plasmid cocktails and the DharmaFECT 1 transfection reagent was employed to transfect mouse keratinocytes with TOMM70 si-RNA and Control si-RNA (100 nM) (Dharmacon) as described previously.^93,94^ Cells and conditioned media were collected 48 h after transfection for further experiments.

### Respirometry Assay

Mitochondrial respiration, glycolysis, and energy phenotypes of murine keratinocytes after the addition of Exo_mϕ_ were measured with a Sea Horse XFe96 Analyzer using a Sea horse XF Cell Mito Stress Test Kit (Agilent; # 103015-100) as described previously.^95^

### JC-1 Assay

The mitochondrial membrane potential of normoxic murine keratinocytes and hypoxic murine keratinocytes were measured using a JC-1 assay kit (Thermo Fisher Scientific; # T3168) as per the manufacturer’s instructions.^95^

### BrdU Cell Proliferation Assay

The cell proliferation assay was studied using an eBioscience BrdU Staining Kit for Flow Cytometry FITC (Thermo Fisher Scientific; # 8811-6600-42) as per the manufacturer’s instructions.

### RNA Extraction and Quantitative Real-Time PCR

RNA was extracted from cells using the miRVana miRNA isolation kit (Thermo Fisher Scientific; # AM1561) according to the manufacturer’s protocol. mRNA was quantified by real-time or quantitative (Q) PCR assay using the PowerUp SYBR Green Master Mix (Thermo Fisher Scientific; # A25742).^96,97^

### Keratinocyte Migration Assay

Murine keratinocyte migration assay was conducted using two-well culture inserts (IBIDI; # 80209) according to the manufacturer’s instructions. Briefly, murine keratinocytes were seeded on two chambers of the insert, and a confluent keratinocyte monolayer was formed within 24 h. Removal of the insert created a gap in the monolayer. After the removal of inserts, d0 and d5 Exo_mϕ_ were added to the murine keratinocytes. Keratinocyte migration was measured and studied using phase-contrast microscopy following withdrawal of the inset for 80 h. In another experiment, following *in vitro* transfection, keratinocytes were seeded on two chambers of insert, and migration was studied (without the addition of exosomes) for 72 h. The extent of migration was analyzed by measuring the gap at 80 and 72 h using ImageJ software.^95^

### Spatial Frequency Domain Imaging (SFDI)

Clarify (Modulim Inc., CA) equipment was used to acquire data for murine tissue oxygenation. The equipment was calibrated using a company-provided pad prior to data acquisition. The mice were anesthetized using isoflurane. The preliminary processing enabled us to obtain oxyhemoglobin (oxy-Hb), and deoxyhemoglobin (deoxy-Hb) maps were obtained along with the other tissue chromophore maps using the built-in software that was used to acquire raw data. The images acquired were postprocessed in MATLAB. This information was then transformed into tissue chromophore concentration using a linear least-squares fit test applied to the NIR data.

### Imaging Mass Cytometry

All antibodies in the panel were initially tested by immunofluorescence and conjugated to metals using the Maxpar Multimetal Labeling Kit (Standard Biotools) according to the manufacturer’s protocol.^98^ Data acquisition was performed on a Helios time-of-flight mass (TOF) cytometer coupled to a Hyperion Imaging System (Standard Biotools). Before laser ablation, optical images of slides were acquired using Hyperion v1.0.560.6 software. Laser ablation was performed at a resolution of approximately 1 mm and a frequency of 200 Hz.^84^

### Data Analysis

The acquired image of the tissue was divided into six segmentations based on structural markers using a deep learning algorithm. The segmented tissue was further subjected to cell segmentation using a deep learning classifier based on DNA1 and DNA2 channels with expansion of the cell body from the nuclei. To avoid noise from neighboring or overlapping cells, we chose a nuclear compartment for cell segmentation. The nuclear compartment provides better differentiation, as only signals closely associated with the cell are considered for analysis. Cell phenotyping was performed by combining the biomarker positivity and the regions determined by the tissue segmentation.

### Immunohistochemistry (IHC), Immunocytochemistry (ICC), and Microscopy

Immunohistochemistry was performed on murine tissues according to the previously reported protocol.^88,93^ Briefly, deparaffinized sections of murine tissues were blocked with 10% normal goat serum and incubated overnight at 4 °C with specific antibodies. Immunostainings of F4/80 (Bio-Rad; # MCA497R; 1:200), K14 (Covance; # PRB-155P-100; 1:400), GFP (Abcam; # ab6556; 1:200), RFP (Rockland Immunochemicals; # 600-901-379; 1:250), TOMM70 (Abcam; # ab289977; 1:1000), OPA1 (Abcam; # ab157457; 1:200), DRP1 (Abcam; # ab184247; 1:500), iNOS (Abcam; # ab115819; 1:100), MPO (Abcam; # ab9535; 1:50), Involucrin (Thermo Fischer Scientific; # PA5-104453; 1:200), Filaggrin (Genetex; # GTX37695; 1:200), E-cadherin (Cell Signaling Technology; # 24E10; 1:400), ZO-1 (Thermo Fischer Scientific; # 61-7300; 1:200), Desmoglein-2 (Abcam; # ab96761; 1:200), Connexin43 (Thermo Fischer Scientific; # CX-1B1(13-8300); 1:100), ZO-2 (Thermo Fischer Scientific; # 38-9100; 1:200), Loricrin (Biolegend; # PRB-145P; 1:200), Occludin (Thermo Fischer Scientific; # 71-1500; 1:200), MCP-1 (eBioscience; # 11-7096-71; 1:100), COX2 (Abcam; # ab15191; 1:100), and MIP-1α (Abcam; # ab25128; 1:200) were performed on paraffin sections of murine skin and WE skin tissue samples. For antibody validation, a rabbit isotype control (Abcam; # ab27478; 1:400) was employed.^99^ Signal was visualized following incubation with fluorescence-tagged (Alexa 488-tagged α-rabbit; 1:200; Alexa 568-tagged α-rabbit; 1:200; Alexa 488-tagged α-chicken; 1:200; Alexa 568-tagged α-rat; 1:200) and counterstained with DAPI. Immunocytochemistry was performed on murine keratinocytes, mϕ, endothelial, and fibroblasts following transfection with plasmid cocktails using an anti-RFP antibody (Rockland Immunochemicals; # 600-901-379; 1:250).^20^ Immunocytochemistry of murine mϕ and keratinocytes was performed using antibodies against RAB5A (Abcam; # ab302987; 1:200), TOMM70 (Abcam; # ab289977; 1:1000), and GFP (Abcam; # ab6556; 1:200). Mitotracker green (Thermo Fisher Scientific; # M7514) was used to label mitochondria. Imaging was done on an Axio Scan (Z1, Zeiss, Germany) and super-resolution confocal system (CARL ZEISS confocal microscope LSM 880). Quantification of the fluorescent intensity was examined using ImageJ software with a colocalization plugin and Zen software (Zen blue 3.1). Mitochondria network morphology analysis was performed from cells stained with Mitotracker red using ImageJ macro toolset (MiNA).^100^

### Histomorphometric Analysis

Wound length, granulation tissue formation, and the length of the epithelial gap were quantitatively determined from hematoxylin and eosin (H&E)-stained paraffin tissue sections using ZEN blue 3.2 (Zeiss) software as described previously.^83,94^

### Tissue Nanotransfection (TNT) 2.0

*In vivo*, TNT was performed using a TNT chip.^87,101^ The *Lyz2*-promotor-driven plasmids cocktail was loaded on the reservoir of the TNT chip and when an electric pulse (at 200 V, 10 pulses, and 10 ms) was applied between the TNT chip and the tissue, the negatively charged plasma DNA traveled from the reservoir to mϕ by electrophoresis and enter them by electroporation.^102,103^ For delivery of *Krt14*-promotor driven “don’t eat me” plasmid cocktail to the keratinocytes, TNT was performed at 100 V.^19^

### Transepidermal Water Loss (TEWL)

To evaluate the skin barrier function *in vivo*, TEWL was measured from the skin and wounds using DermaLab TEWL Probe (cyberDERM, Broomall, PA).^86,94^ The data were expressed in gm^–2^ h^–1^.

## Quantification and Statistical Analysis

### PCA Analysis

A multivariate principal component analysis (PCA) technique was carried out using the OriginPro PCA spectroscopy app for Raman spectra investigation.

### Statistical Analysis

GraphPad Prism (GraphPad Software) version 9.0 was used for statistical analyses. The ΔΔCt value was used for the statistical analysis of all RT-qPCR data. Statistical analysis between multiple groups was performed using a one-way analysis of variance with the *posthoc* Bonferroni multiple comparison test. Statistical analysis between two groups was performed using unpaired Student’s two-sided *t*-tests. *P* < 0.05 was considered statistically significant. Significance levels and exact *P* values were indicated in all relevant figures. Data were checked for normality prior to analysis. Data for independent experiments were presented as means ± SEM unless otherwise stated.

## Supporting Information Available

Cell phenotyping markers (ST1); GEO (GSE165816) data set sample details (ST2); observed Raman peaks (ST3); observed FTIR peaks (ST4); labeling of Exo_mϕ_ in murine skin (S1); keratinocyte–macrophage crosstalk in human diabetic foot ulcer (DFU) samples (S2); characterization of Exo_mϕ_ from murine WE tissue (S3); TOMM70 in WE keratinocytes is susceptible to hypoxia (S4); crosstalk between resident keratinocytes and WE mϕ is critical for functional wound closure (S5); compromised uptake of Exo_κ_ by WE mϕ results in impaired epidermal junctional proteins (S6); compromised uptake of Exo_κ_ by WE mϕ results in impaired epidermal differentiation (S7); expression of TOMM70 in the leading-edge epidermis post wounding (S8) (PDF)
Resources availability (materials, data and code), MATLAB code for the quantification of tissue StO_2_ (SI1) (PDF)
Live-cell confocal images showing uptake of Exo_κ_ by mouse mϕ using LSM 880, K14-tetsp (Movie S1) (MP4)
Live-cell confocal images showing uptake of Exo_κ_ by mouse mϕ using LSM 880, K14-tetsp-CD47 (Movie S2) (MP4)
