# Supplementary material for: Mitochondrial Bioenergetics of Functional Wound Closure is Dependent on Macrophage–Keratinocyte Exosomal Crosstalk
Source: ACS Nano. 2024 Oct 25;18(44):30405–20. doi: 10.1021/acsnano.4c07610 (PMC11544725; doi:10.1021/acsnano.4c07610)
Supplement: Supplementary file 1 — nn4c07610_si_001.pdf [file nn4c07610_si_001.pdf]

The Mitochondrial Bioenergetics of Functional Wound Closure is Dependent on Macrophage-Keratinocyte Exosomal Crosstalk

*Anu Sharma[1], Rajneesh Srivastava[1], Surya C. Gnyawali[1], Pramod Bhasme[1], Adam J. Anthony[2], Yi Xuan[1], Jonathan C. Trinidad[2], Chandan K. Sen[1], David E. Clemmer[2], Sashwati Roy[1], Subhadip Ghatak[1]**

[1]McGowan Institute for Regenerative Medicine, Department of Surgery, University of Pittsburgh, PA, 15219, USA

[2]Department of Chemistry, Indiana University, Bloomington, IN, 47405, USA

*** Correspondence:** ghataks@pitt.edu

Supplementary Table 1. Cell phenotyping markers

Phenotype	Marker	Regions selected
Epithelial cells	E-cadherin^{+}, Pan-cytokeratin^{+}	Epidermis, dermis, and hypodermis
Hair follicle		Hair follicle
Myeloid cells	F4/80^{+}, CD14^{+}, CD11c^{-}	Epidermis, dermis, and hypodermis
Smooth muscle cells	αSMA^{+}, Vimentin^{-}	Epidermis, dermis, and hypodermis
Endothelial cells	CD31^{+}	Epidermis, dermis, and hypodermis
Fibroblast	Vimentin^{+}, αSMA^{+}	Epidermis, dermis, and hypodermis
Connective tissue	Col1A2^{+}	Dermis and hypodermis
Adipose tissue		Adipose tissue

Supplementary Table 2. GEO (GSE165816) dataset sample details

Patient ID	Samples	Library ID	Code	# Cells
P1	GSM5050530	G6	NHDFU	1885
P2	GSM5050533	G9	NHDFU	3674
P3	GSM5050557	G33	NHDFU	3266
P3	GSM5050558	G34	NHDFU	2946
P4	GSM5050563	G39	NHDFU	1731
P5	GSM5050531	G7	HDFU	1438
P5	GSM5050532	G8	HDFU	2047
P6	GSM5050573	G49	HDFU	1880
P7	GSM5050566	G42	HDFU	1143
P8	GSM5050569	G45	HDFU	2171
P9	GSM5050547	G23	HDFU	4181
P9	GSM5050527	G4	HDFU	2297
P10	GSM5050523	G2	HDFU	3093
P11	GSM5050539	G15	HDFU	2062

Supplementary Table 3. Observed Raman peaks

**Wavenumber (cm^{-1})**	**d0 $Exo_{m\phi}$**	**d5 $Exo_{m\phi}$**	**Origin**
650	No	Yes	Proteins
750	Yes	No	O-P-O stretching in nucleic acids
800	No	Yes	Proteins
930-950	Yes	Shifted	Nucleic acids
1070	Yes	Yes	Lipids, proteins
1147	No	Yes	Proteins
1200-1300	No	Yes	Amide III
1300-1400	Shifted	Yes	Proteins
1400-1460	No	Yes	Nucleic acids, proteins, and lipids
1530	Yes	No	Proteins, nucleic acids
1600-1700	Slight peak	No	Amide I, unsaturated lipids
2900	Less intense	More intense	Proteins, lipids

Supplementary Table 4. Observed FTIR peaks

**Wavenumber (cm^{-1})**	**d0 $Exo_{m\phi}$**	**d5 $Exo_{m\phi}$**	**Origin**
700	No	Yes	Proteins
1000	No	Yes	O-P-O stretching in nucleic acids
1600	Narrow	Little broader	Phosphodiester
2800	No	Yes	Nucleic acids, proteins, and lipids
3400	Narrow peak	Broad peak	Amide I, unsaturated lipids

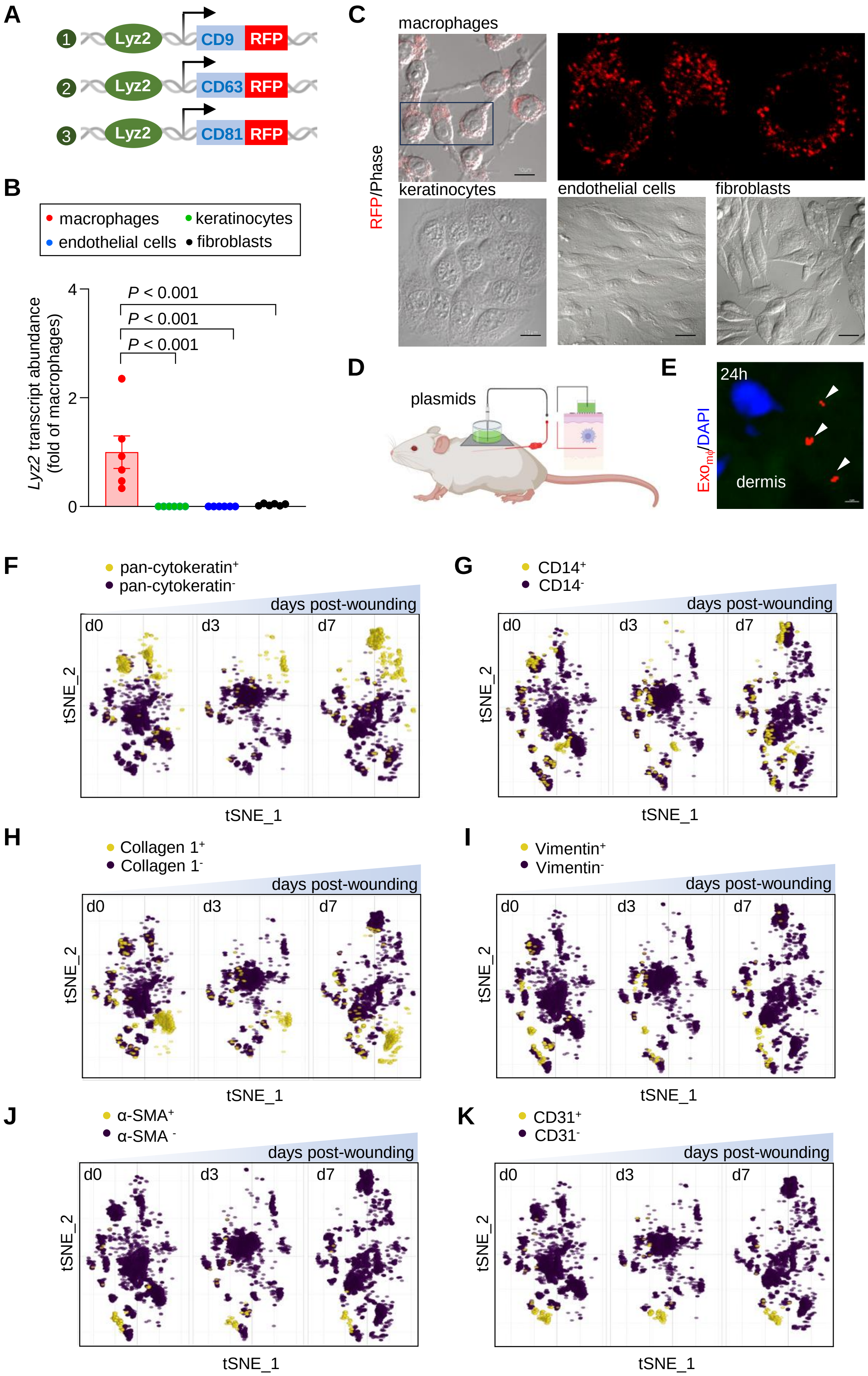

Supplementary Figure 1

Figure S1: Labelling of Exo$_{m\phi}$ in murine skin. **A,** Plasmid design with Lyz2 promoter-driven recombinant plasmids encoding CD9, CD63, or CD81 with “in frame” RFP reporter. **B,** Transcript abundance of *Lyz2* in murine dermal epithelial cells, dermal microvascular endothelial cells, dermal adult fibroblasts, and mϕ. (n=6). **C,** Representative confocal microscopic images showing expression of RFP (red) in murine dermal epithelial cells (keratinocytes), dermal microvascular endothelial cells, dermal adult fibroblasts, and mϕ. Scale, 10 µm. **D,** Schematic diagram showing the delivery of the three *Lyz2*-promoter driven plasmids *via* TNT on dorsal murine skin. **E,** Super-resolution confocal microscopic image showing RFP-labelled Exo$_{m\phi}$ (red) in the dermis. Scale, 2 µm. **F-K,** tSNE-plot with a binary color bar showing the abundance of pan-cytokeratin$^+$, CD14$^+$, Collagen 1$^+$, Vimentin$^+$, α-SMA$^+$, and CD31$^+$ cell distributions at d0, d3 and d7 post-wounding. Data in **B** were shown as mean ± SEM and were analyzed by one-way ANOVA with the *post-hoc* Bonferroni’s multiple comparison test.

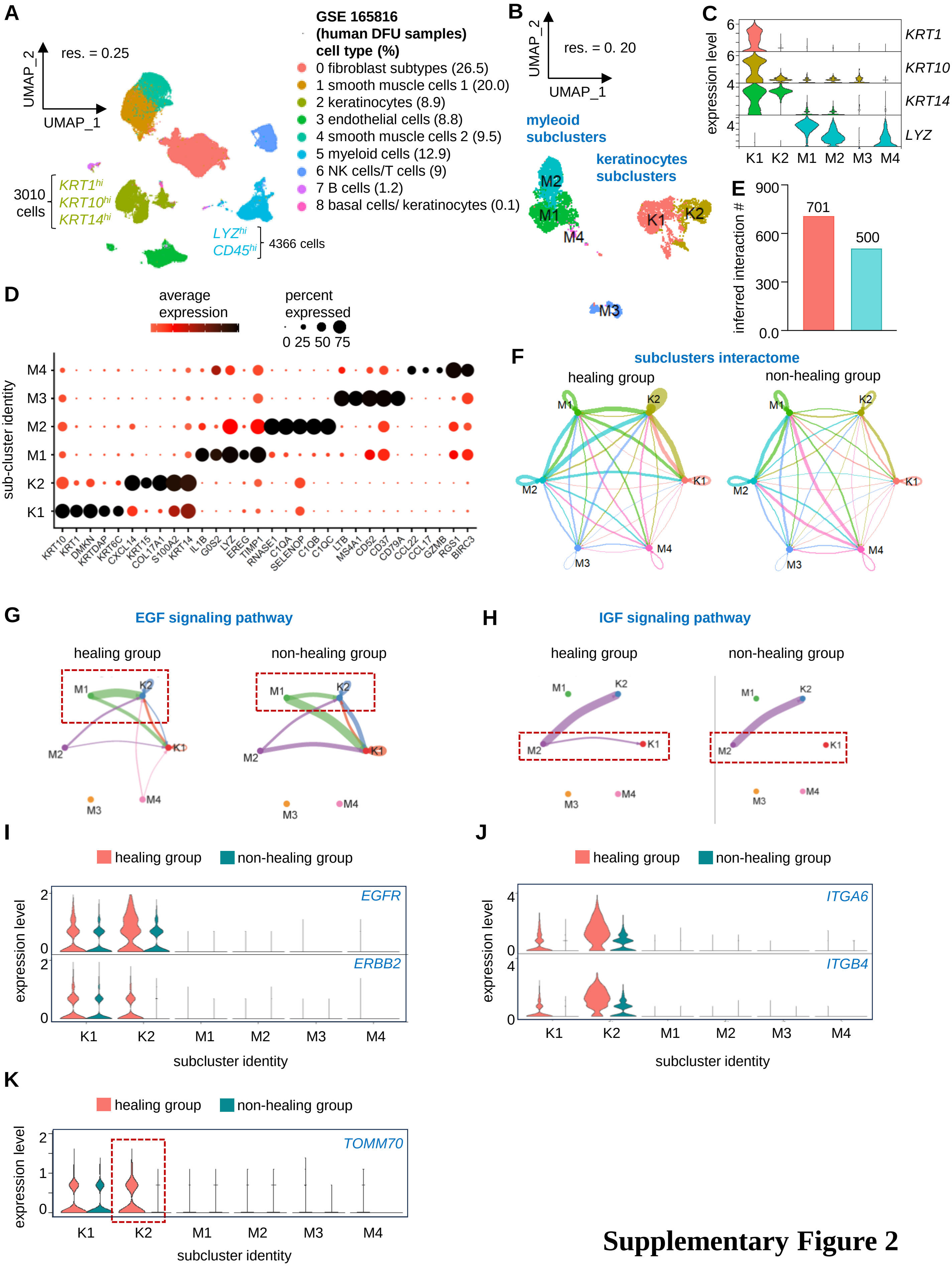

Supplementary Figure 2

Figure S2: Keratinocyte-mϕ crosstalk in human diabetic foot ulcers (DFU) samples. Single-cell RNA seq data of 14 human DFU samples from eleven diabetic subjects with healing (n=9) and non-healing (n=5) status, were downloaded from Gene Expression Omnibus (GEO) with accession number GSE165816 (Table S2). Transcriptomic scRNA-seq data was quality filtered, normalized using 'SCT-transformation' and stored as Seurat objects in R. Further, these Seurat objects were integrated and executed for clustering analysis (with resolution 0.20) using Seurat (See methods) **A,** Cluster of cells derived from this dataset were illustrated in UMAP plot, showing the 9 (0-8) clusters with distinct cell type annotations, based on known signature genes and their proportions. **B,** Keratinocytes and myeloid cell subclusters identified by re-clustering analysis (see methods), were illustrated as UMAP plot. **C,** Expression profile of known signature genes of keratinocytes and myeloid cells in respective subtypes was illustrated in violin plot. **D,** Dot plot showing the average log2 expression of top 5 marker genes of each sub cluster where the size of each dot represents the percentage of expressed cells and darker color scale indicate high expression. **E,** Bar graph showing the total number of cell-cell interactions observed in healing and non-healing groups using CellChat package in R. **F,** The inferred cell-cell communication networks for each group was illustrated in circle-net plot, where node representing the marked subcluster, color matched with connecting edges indicate outgoing signals and width of edges represent increased (or decreased) signaling with participating node in the connectome.

G-H, Proportion plot illustrating the relative information flow (see methods) of significantly enriched signaling pathways between the healing and non-healing groups. Inferred communication for EGF signaling and IGF signaling observed in each group was illustrated in circle-net plots, where nodes and connecting edges indicate the signals of participating subclusters. **I-K,** Average log2 expression profile of *EGF*, *IGF* signaling specific genes, and *TOMM70* were shown as violin plots.

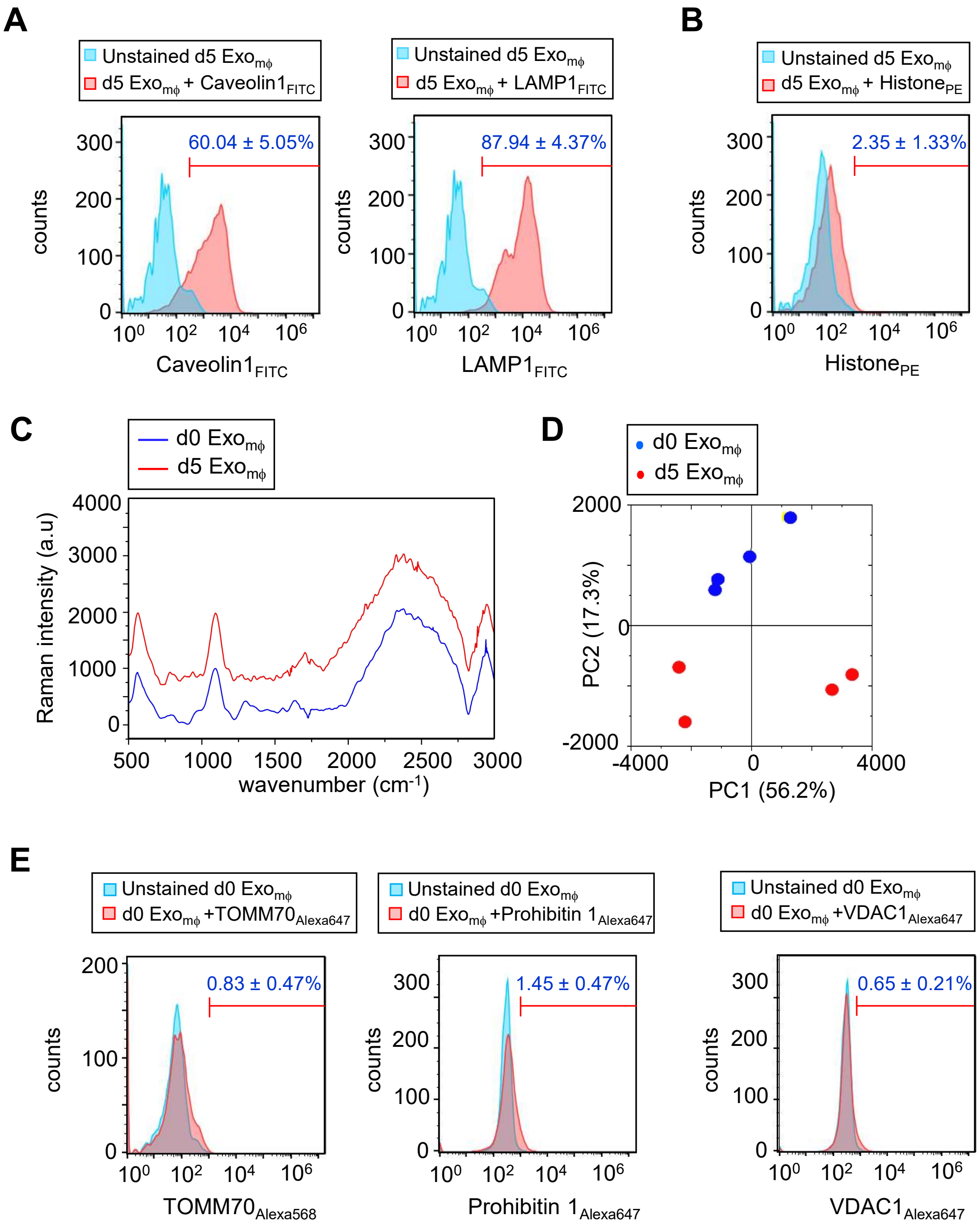

Supplementary Figure 3

Figure S3: Characterization of $Exo_{m\phi}$ from murine WE tissue. A-B, Bead flow cytometric analysis of murine $Exo_{m\phi}$ conjugated with supermagnetic dynabeads functionalized with RFP antibody showing binding of Caveolin-1_{FITC}, $LAMP1_{FITC}$ and $Histone_{PE}$ antibodies (contaminants associated with the exosomes). The histogram demonstrates the shift in FITC and PE fluorescence after binding with the antibodies. The mean percentage of beads with $Exo_{m\phi}$ was mentioned over the marker bar. **C,** Representative Raman spectra of $Exo_{m\phi}$ isolated from murine skin and d5 WE tissue. **D,** 2D-score plot constructed from principal component analysis of Raman spectra of $Exo_{m\phi}$ isolated from murine skin and d5 WE tissue. (n=4). **E**, Bead flow cytometric analysis of murine d0 $Exo_{m\phi}$ conjugated with supermagnetic dynabeads functionalized with RFP antibody showing binding of $TOMM70_{AF568}$, Prohibitin 1_{AF647}, and $VDAC1_{AF647}$ antibodies. The histogram demonstrates the shift in fluorescence after binding with the antibodies. The mean percentage of beads with $Exo_{m\phi}$ was mentioned over the marker bar. Data are representative of three independent experiments.

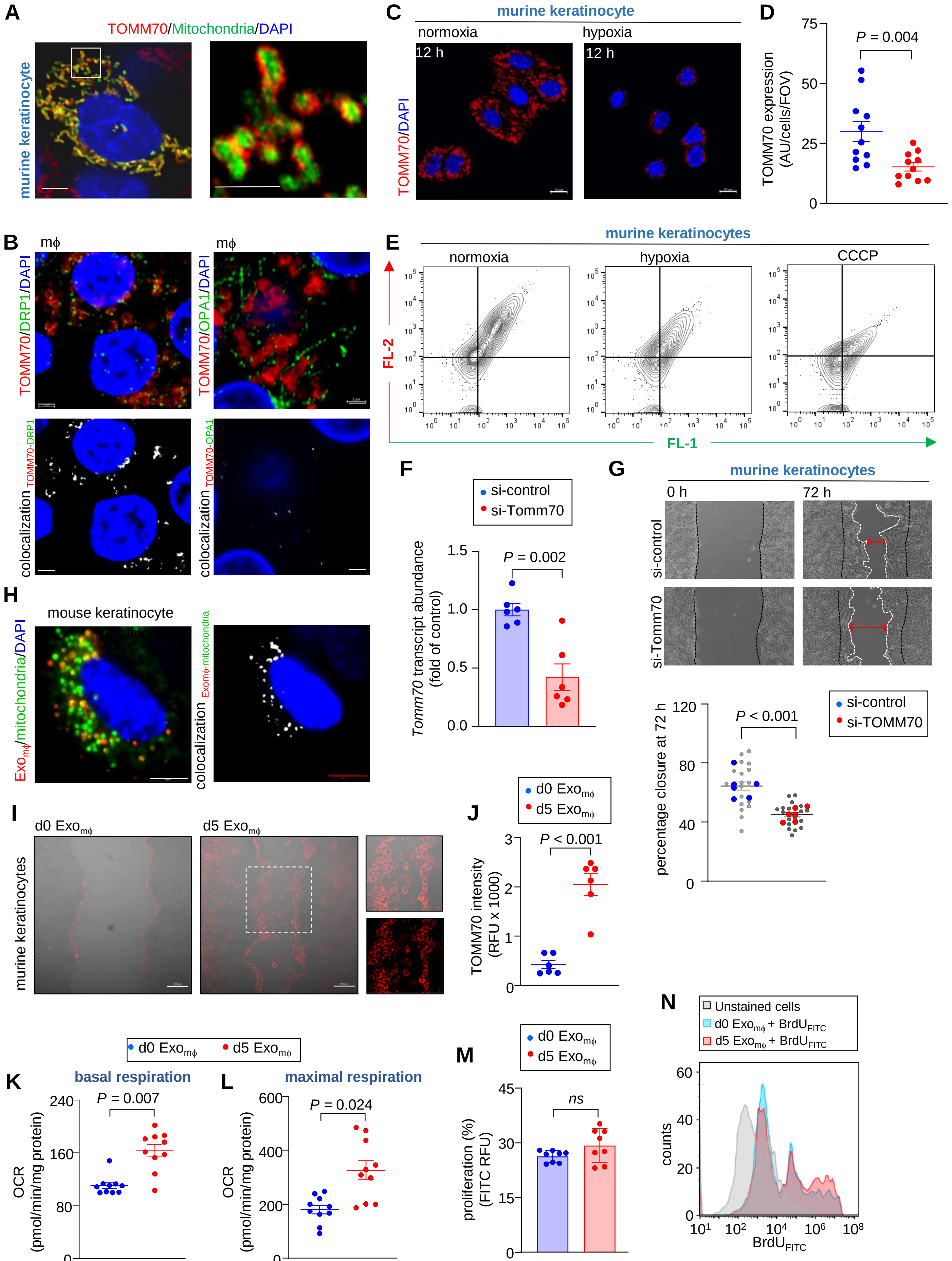

Supplementary Figure 4

Figure S4: TOMM70 in WE keratinocytes is susceptible to hypoxia. A, Representative immunocytochemistry of TOMM70 (red) costained with mitotracker (green) and DAPI counterstaining in mouse keratinocytes under normoxic conditions. Scale, 2 μm and 1 μm (inset). **B,** Representative immunocytochemistry of DRP1 and OPA1 costained with TOMM70 and DAPI counterstaining in mouse mϕ. Scale, 2 μm. The co-localization of DRP1 and OPA1 with TOMM70 is shown in white. **C-D,** Representative immunocytochemistry and quantification of TOMM70 with DAPI counterstaining in mouse keratinocytes under normoxia and hypoxia for 12 h. (n=11). Scale, 10 μm. **E,** Flow cytometry analysis of mitochondrial membrane potential using JC-1 in mouse keratinocyte under normoxia and 12 h hypoxia. Cells exposed to carbonyl cyanide m-chlorophenyl hydrazine were used as a positive control. **F,** Transcript abundance of *Tomm70* in mouse keratinocytes after transfection with si-Tomm70. (n=6). **G,** Representative phase contrast microscopic images of murine keratinocytes at 0 h and 72 h showing migration following transfection with si-Tomm70 and its respective control. Keratinocyte migrations were quantified and expressed as percentage closure. For each image, the distance of the migrating front at the top middle, and bottom were measured (grey dots) and the mean value was plotted (blue, red). (n=6). Scale 200 μm. **H,** Representative immunocytochemistry of RFP as a marker $Exo_{m\phi}$ (red) co-stained with mitotracker (green) and DAPI counterstaining in mouse keratinocytes demonstrating the fusion of $Exo_{m\phi}$ with keratinocytes mitochondria under hypoxic conditions. Scale, 5 μm. Colocalization of $Exo_{m\phi}$ with the mitochondria in keratinocytes was shown in white.

I-J, Representative fluorescence and DIC images of murine keratinocytes at 80 h after migration and quantification of TOMM70 abundance. Cells were fixed for immunocytochemistry. (n=6). Scale, 100 µm. **K,** Basal mitochondrial respiration in mouse keratinocytes as calculated from OCR. (n=10). **L,** Basal mitochondrial respiration in mouse keratinocytes as calculated from OCR. (n=10). **M-N,** Flow cytometry analysis and quantification of murine keratinocytes following incubation with d0 $Exo_{m\phi}$ and d5 $Exo_{m\phi}$ using BrdU staining cell proliferation kit. Cells without BrdU were used as a negative control. Data in **D**, **F**, **H**, **J**, **K**, **L,** and **M** were shown as mean ± SEM and were analyzed by two-tailed unpaired Student's *t-test*.

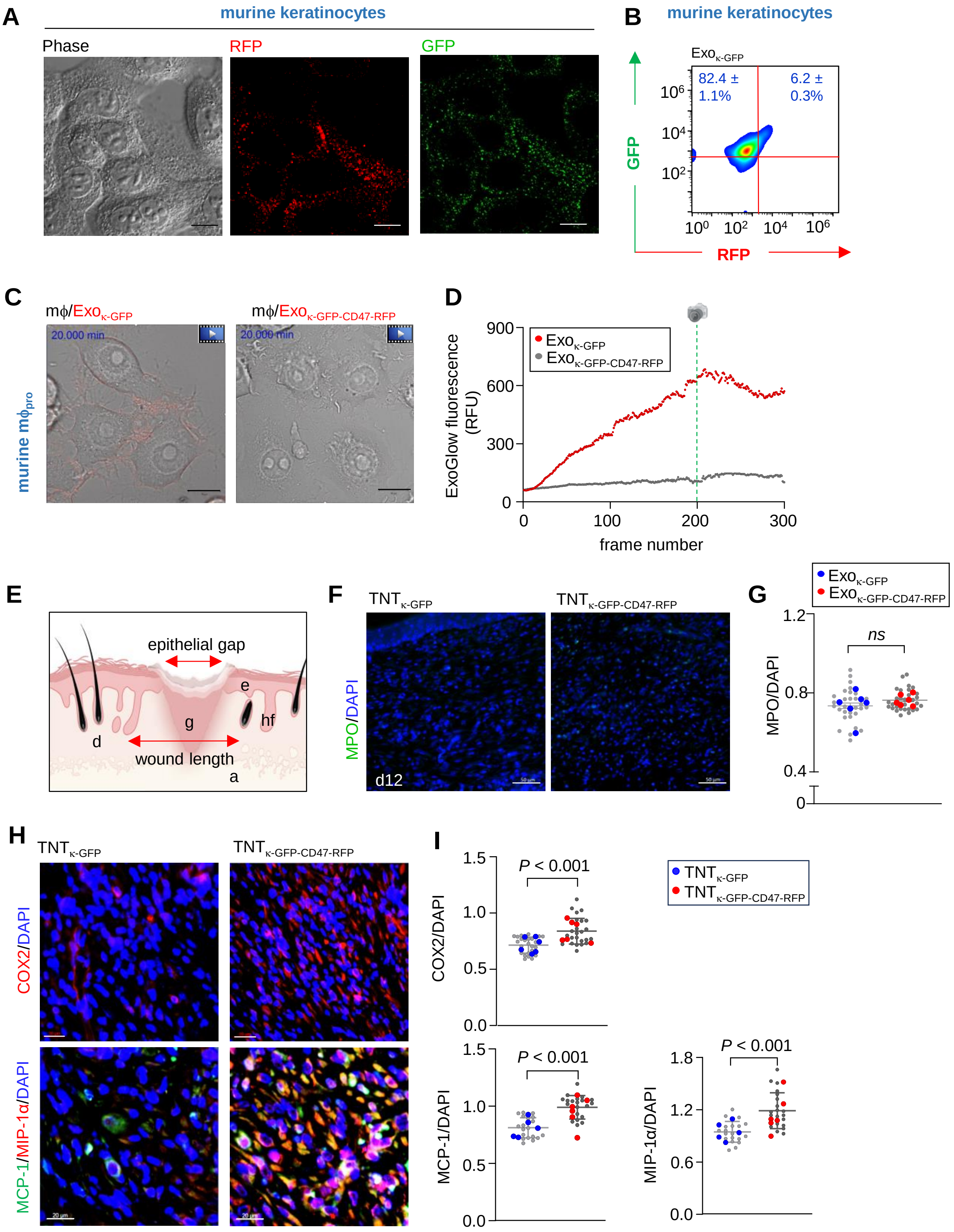

Supplementary Figure 5

Figure S5: Crosstalk between resident keratinocytes and WE mϕ is critical for functional wound closure. A, Representative confocal images showing co-expression of RFP (red) and GFP (green) and DIC image of mouse keratinocytes. Scale, 20 µm. **B,** Flow cytometric analysis of murine Exo$_{\kappa}$ on pan CD magnetic beads showing GFP fluorescence. **C,** Live-cell confocal images showing compromised uptake of Exo$_{\kappa\text{-GFP-CD47-RFP}}$ by d3 proinflammatory mϕ. Scale, 10 µm. Both Exo$_{\kappa\text{-GFP}}$ and Exo$_{\kappa\text{-GFP-CD47-RFP}}$ were stained with ExoGlow before imaging. Indicate movies in the supplement. **D,** Quantification of ExoGlow relative fluorescence intensity with time. **E,** Schematic diagram showing the epithelial gap and wound length. **F,** Representative co-immunofluorescence staining of MPO (green) and DAPI counterstaining in WE tissue at d12 post wounding in C57BL/6 mice. Scale, 50 µm. **G,** Quantification of MPO intensity in WE tissue at d12 post wounding. Each corresponds to one quantified ROI, except the blue and red dots, which correspond to the mean of each mouse. (n = 6). **H,** Representative co-immunofluorescence staining of COX2 (red), MCP-1 (green), and MIP-1α (red) and DAPI counterstaining WE tissue at d12 post wounding in C57BL/6 mice. Scale, 20 µm. **I,** Quantification of COX2, MCP-1, and MIP-1α intensity in WE tissue at d12 post wounding. Each corresponds to one quantified ROI, except the blue and red dots, which correspond to the mean of each mouse. (n=5-6). Data in **G** and **I** were shown as mean ± SEM and were analyzed by two-tailed unpaired Student's *t-test*.

A

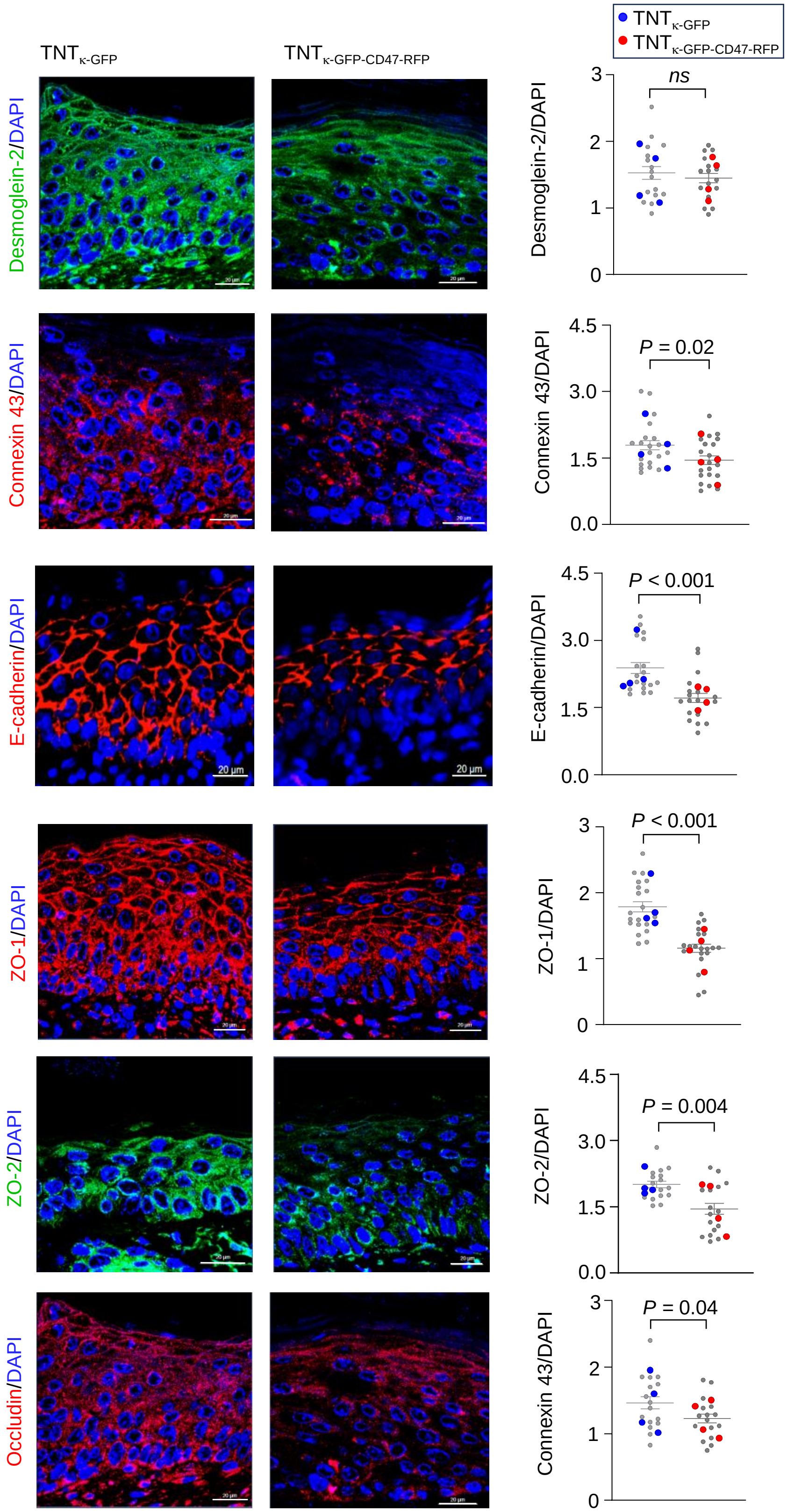

Supplementary Figure 6

Figure S6: Compromised uptake of Exo_{κ} by WE mϕ results in impaired epidermal junctional proteins. A, Representative confocal images showing expression of Desmoglein-2 (green), Connexin 43 (red), E-cadherin (red), ZO-1 (red), ZO-2 (green), and Occludin (red) with DAPI counterstaining in WE tissue at d12 post wounding in C57BL/6 mice with either $Exo_{\kappa\text{-GFP}}$ and $Exo_{\kappa\text{-GFP-CD47-RFP}}$. Scale, 20 μm. Each dot corresponds to one quantified ROI, except the blue and red dots, which correspond to the mean of each mouse. At least 4 ROI per mouse (n=4). Data were expressed as mean ± SEM and were analyzed by two-tailed unpaired Student's *t-test*.

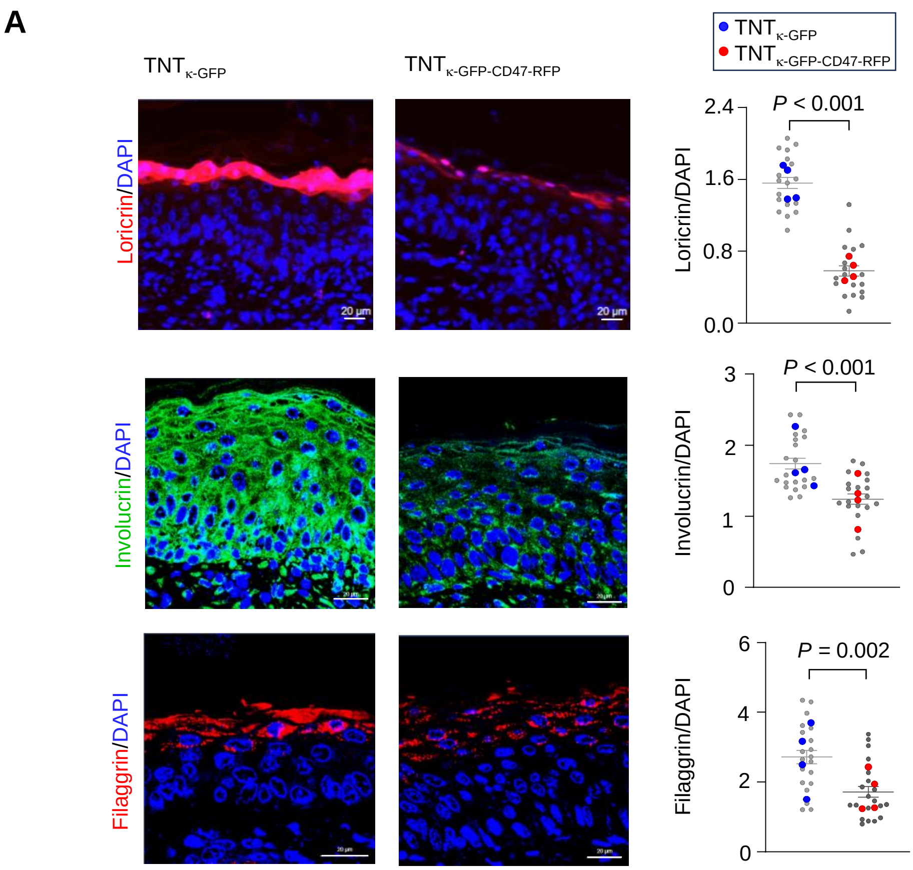

Figure S7: Compromised uptake of Exo$_{\kappa}$ by WE mϕ results in impaired epidermal differentiation. A, Representative confocal images showing expression of Loricrin (red), Involucrin (green), and Filaggrin (red) with DAPI counterstaining in WE tissue at d12 post wounding in C57BL/6 mice with either Exo $_{\text{-GFP}}$ and Exo $_{\text{-GFP-CD47-RFP}}$. Scale, 20 µm. Each dot corresponds to one quantified ROI, except the blue and red dots, which correspond to the mean of each mouse. At least 4 ROI per mouse (n=4). Data were expressed as mean ± SEM and were analyzed by two-tailed unpaired Student's *t-test*.

Supplementary Figure 7

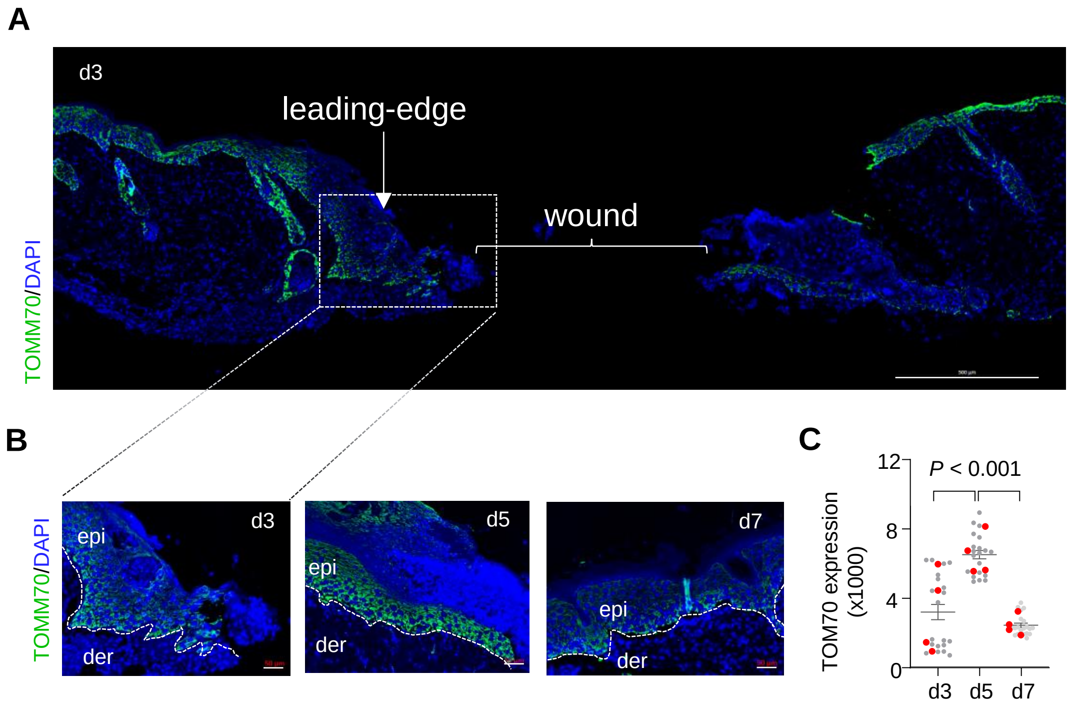

Figure S8: Expression of TOMM70 in the leading-edge epidermis post wounding. A, The d3 WE tissue section shows the wound and the leading-edge epidermis. **B,** Representative co-immunofluorescence staining of TOMM70 (green) with DAPI counterstaining in WE tissue at d3, d5, and d7 post wounding in C57BL/6 mice. White dashed lines indicate the dermal-epidermal junction. Scale, 50 μm. **C,** Quantification of TOMM70 intensity in WE tissue at d3, d5, and d7 post wounding. Each corresponds to one quantified ROI, except the blue and red dots, which correspond to the mean of each mouse. (n=4). Data in **C** were shown as mean ± SEM and were analyzed by one-way ANOVA with the *post-hoc* Bonferroni's multiple comparison test.

Supplementary Figure 8
