# Supplementary material for: Mitochondrial Bioenergetics of Functional Wound Closure is Dependent on Macrophage–Keratinocyte Exosomal Crosstalk
Source: ACS Nano. 2024 Oct 25;18(44):30405–20. doi: 10.1021/acsnano.4c07610 (PMC11544725; doi:10.1021/acsnano.4c07610)
Supplement: Supplementary file 2 — nn4c07610_si_002.pdf [file nn4c07610_si_002.pdf]

The Mitochondrial Bioenergetics of Functional Wound Closure is Dependent on Macrophage-Keratinocyte Exosomal Crosstalk

Anu Sharma[1], Rajneesh Srivastava[1], Surya C. Gnyawali[1], Pramod Bhasme[1],

Adam J. Anthony[2], Yi Xuan[1], Jonathan C. Trinidad[2],

*Chandan K. Sen[1], David E. Clemmer[2], Sashwati Roy[1], Subhadip Ghatak[1]**

[1]McGowan Institute for Regenerative Medicine, Department of Surgery, University of Pittsburgh, PA, 15219, USA

[2]Department of Chemistry, Indiana University, Bloomington, IN, 47405, USA

*** Correspondence:** ghataks@pitt.edu

RESOURCES AVAILABILITY

Lead contact

Further information and requests for resources and reagents should be directed to and will be fulfilled by the lead contact, Subhadip Ghatak (ghataks@pitt.edu).

Materials availability

The Lyz2 promoter-driven reporter plasmids and the KRT14 promoter-driven “don’t eat me” plasmids generated in this study are available from the lead contact upon request.

Data and code availability

- All raw data is available from the lead contact upon request.
- The original MATLAB code for imaging tissue StO_2 concentration is provided in supplemental item 2.
- R code for single-cell RNA sequencing data processing analysis is available at https://github.com/RajneeshSrivastava/MQ-KR-crosstalk
- Any additional information required to reanalyze the data reported in this paper is available from the lead contact upon request.

Supplemental Information 1. MATLAB code for quantification of tissue StO_2

```
% Demo to have the user freehand draw an irregular shape over
% a gray scale image, have it extract only that part to a new image,
% and to calculate the mean intensity value of the image within that shape.
% Coded by Dr. Surya C. Gnyawali, PhD
if(~isdeployed)
  cd(fileparts(which(mfilename)));
end
clc;
clear;
close all;
imtool close all;
workspace;  % the workspace panel is showing.
fontSize = 16;
% standard MATLAB gray scale demo image.
grayImage = imread('EnhancedImageHb.jpg');
subplot(2, 3, 1);
imshow(grayImage, []);
title('Original Image', 'FontSize', fontSize);
set(gcf, 'Position', get(0,'Screensize')); % Maximize figure.
message = sprintf('Left click and hold to begin drawing.\nSimply lift the mouse button to
finish');
uiwait(msgbox(message));
hFH = imfreehand();
% Create a binary image ("mask") from the ROI object.
binaryImage = hFH.createMask();
% Display the freehand mask.
subplot(2, 3, 2);
imshow(binaryImage);
title('Binary mask of the region', 'FontSize', fontSize);
% Calculate the area, in pixels, that they drew.
numberOfPixels1 = sum(binaryImage(:))
% Another way to calculate it that takes fractional pixels into account.
numberOfPixels2 = bwarea(binaryImage)
% Get coordinates of the boundary of the freehand drawn region.
structBoundaries = bwboundaries(binaryImage);
xy=structBoundaries{1}; % Get n by 2 array of x,y coordinates.
x = xy(:, 2); % Columns.
y = xy(:, 1); % Rows.
subplot(2, 3, 1); % Plot over original image.
hold on; % Don't blow away the image.
plot(x, y, 'LineWidth', 2);
% Burn line into image by setting it to 255 wherever the mask is true.
burnedImage = grayImage;
burnedImage(binaryImage) = 255;
% Display the image with the mask "burned in."
subplot(2, 3, 3);
imshow(burnedImage);
```

```
caption = sprintf('New image with\nmask burned into image');
title(caption, 'FontSize', fontSize);
% Mask the image and display it.
% Will keep only the part of the image that's inside the mask, zero outside mask.
blackMaskedImage = grayImage;
blackMaskedImage(~binaryImage) = 0;
subplot(2, 3, 4);
imshow(blackMaskedImage);
title('Masked Outside Region', 'FontSize', fontSize);
% Calculate the mean
meanGL = mean(blackMaskedImage(binaryImage));
% Report results.
message = sprintf('Mean value within drawn area = %.3f\nNumber of pixels = %d\nArea in
pixels = %.2f', ...
meanGL, numberOfPixels1, numberOfPixels2);
msgbox(message);
% Now do the same but blacken inside the region.
insideMasked = grayImage;
insideMasked(binaryImage) = 0;
subplot(2, 3, 5);
imshow(insideMasked);
title('Masked Inside Region', 'FontSize', fontSize);
% Now crop the image.
topLine = min(x);
bottomLine = max(x);
leftColumn = min(y);
rightColumn = max(y);
width = bottomLine - topLine + 1;
height = rightColumn - leftColumn + 1;
croppedImage = imcrop(blackMaskedImage, [topLine, leftColumn, width, height]);
% Display cropped image.
subplot(2, 3, 6);
imshow(croppedImage);
title('Cropped Image', 'FontSize', fontSize);
%end
```

RESULTS LIST STUDY TREE

Details	EV-TRACK ID	Experiment nr.	Species	Sample type	Separation protocol	First author	Year	EV-METRIC
+	EV220292	1/4	Mus musculus	NA	(d)(U)C<br>IAF	Sharma, Anu	NA	100%
+	EV220292	2/4	Mus musculus	NA	(d)(U)C<br>IAF	Sharma, Anu	NA	100%
+	EV220292	3/4	Mus musculus	NA	(d)(U)C<br>IAF	Sharma, Anu	NA	100%
+	EV220292	4/4	Mus musculus	NA	(d)(U)C<br>IAF	Sharma, Anu	NA	100%
